# *Plasmodium falciparum* parasite population structure and gene flow associated to anti-malarial drugs resistance in Cambodia

**DOI:** 10.1186/s12936-016-1370-y

**Published:** 2016-06-14

**Authors:** Ankit Dwivedi, Nimol Khim, Christelle Reynes, Patrice Ravel, Laurence Ma, Magali Tichit, Christiane Bourchier, Saorin Kim, Dany Dourng, Chanra Khean, Pheaktra Chim, Sovannaroth Siv, Roger Frutos, Dysoley Lek, Odile Mercereau-Puijalon, Frédéric Ariey, Didier Menard, Emmanuel Cornillot

**Affiliations:** Institut de Biologie Computationnelle (IBC), Montpellier, France; IRCM-INSERM U1194, Institut de Recherche en Cancérologie de Montpellier, Montpellier, France; Université de Montpellier, Montpellier, France; ICM, Institut régional du Cancer Montpellier, Montpellier, France; Malaria Molecular Epidemiology Unit, Institut Pasteur in Cambodia, Phnom Penh, Cambodia; Laboratoire de Biostatistiques, Informatique et Physique Pharmaceutique, UFR Pharmacie, Université de Montpellier, Montpellier, France; Institut de Génomique Fonctionnelle, Montpellier, France; Genopole Sequencing Platform, Institut Pasteur, Paris, France; National Centre for Parasitology, Entomology, and Malaria Control, Phnom Penh, Cambodia; UMR 17, Intertryp, Cirad-IRD, Campus International de Baillarguet, Montpellier, France; IES-UMR 5214, Institut d’Electronique et des Systèmes, Université de Montpellier-CNRS, Montpellier, France; Parasite Molecular Immunology Unit, Institut Pasteur, Paris, France

## Abstract

**Background:**

Western Cambodia is recognized as the epicentre of emergence of *Plasmodium falciparum* multi-drug resistance. The emergence of artemisinin resistance has been observed in this area since 2008–2009 and molecular signatures associated to artemisinin resistance have been characterized in *k13* gene. At present, one of the major threats faced, is the possible spread of Asian artemisinin resistant parasites over the world threatening millions of people and jeopardizing malaria elimination programme efforts. To anticipate the diffusion of artemisinin resistance, the identification of the *P. falciparum* population structure and the gene flow among the parasite population in Cambodia are essential.

**Methods:**

To this end, a mid-throughput PCR-LDR-FMA approach based on LUMINEX technology was developed to screen for genetic barcode in 533 blood samples collected in 2010–2011 from 16 health centres in malaria endemics areas in Cambodia.

**Results:**

Based on successful typing of 282 samples, subpopulations were characterized along the borders of the country. Each 11-loci barcode provides evidence supporting allele distribution gradient related to subpopulations and gene flow. The 11-loci barcode successfully identifies recently emerging parasite subpopulations in western Cambodia that are associated with the C580Y dominant allele for artemisinin resistance in *k13* gene. A subpopulation was identified in northern Cambodia that was associated to artemisinin (R539T resistant allele of *k13* gene) and mefloquine resistance.

**Conclusions:**

The gene flow between these subpopulations might have driven the spread of artemisinin resistance over Cambodia.

**Electronic supplementary material:**

The online version of this article (doi:10.1186/s12936-016-1370-y) contains supplementary material, which is available to authorized users.

## Background

*Plasmodium falciparum* malaria is one of the most severe and wide spread parasitic disease affecting millions of humans in the world. Following the emergence and spread of multidrug resistant parasites is a major challenge. The Cambodian–Thai border is recognized as the epicentre of the emerging resistances. *Plasmodium falciparum* clinical malaria resistance to chloroquine was first documented in 1957 [1, 2] in this area. Later, in 1967 pyrimethamine resistance was also reported in the same region [3, 4]. Molecular epidemiological studies have confirmed that the spread of resistant parasites to these two drugs to Africa has originated from Southeast Asia [5]. In 1990s, mefloquine resistance was consequently observed in this area and more recently, the emergence of artemisinin derivatives resistance was observed along Cambodian-Thai border [6, 7]. The reasons supporting the emergence of multidrug resistance parasites in this area are unknown. Recently, whole genome sequencing data demonstrated that *P. falciparum* populations were highly fragmented in Cambodia [8, 9]. Four subpopulations (KH1, KH2, KH3 and KH4) and one large admixed subpopulation (KHA) were described using samples isolated in the time period 2007–2011 [8]. KH1 subpopulation was shown to be as the ancestral population. The KH2, KH3 and KH4 subpopulations were associated to clinical artemisinin resistance defined by a delayed of parasite clearance in the first 3 days of artesunate monotherapy or artemisinin-based combination therapy (ACT) [8] and were later confirmed to be associated to mutations in the propeller domain of the *Kelch* gene (PF3D7_1343700) located on the chromosome 13 (*k13*) [6]. In their report, Ariey and collaborators clearly showed that the prevalence of mutant *k13* alleles, involved in artemisinin resistance, was much higher in western Cambodian provinces than in eastern Cambodia [6].

In this context, the present study aimed at evaluating the structure of the parasite population at a country-wide scale. Indeed, one hypothesis is that the structure of the parasite population plays an important role in the spread of *k13* mutant alleles from west to east Cambodia. The parasite population structure can be assessed by following different genetic variations such as single nucleotide polymorphisms (SNPs), microsatellite repeats, insertions/deletions and range of gene duplication events [10]. Several molecular approaches have been developed to accurately detect reliable SNPs in the *P. falciparum* genome. For instance, a 24-SNP barcode detected by a robust TaqMan genotyping approach was described by Daniels et al. [11]. Their analysis was performed on African and Thai isolates. At present, novel, rapid and reliable techniques based on fluorescent magnetic beads, such as the LUMINEX technology, have been developed to detect specific alleles. A rapid assay of *Plasmodium* typing was developed using fluorescent microspheres [12]. This assay combined a PCR and a ligation reaction: PCR-LDR-FMA (PCR-based ligase detection reaction-fluorescent microsphere assay).

This paper describes the implementation of the PCR-LDR-FMA for the detection of an 11-SNP barcode. The presence of parasite subpopulations was evaluated and intensive gene flow over Cambodia was described to assess the spread of drug resistance. Of note, a new subpopulation was defined, highly prevalent in northern Cambodia and associated with in vitro mefloquine resistance (expressed by high mefloquine IC_50_ values).

## Methods

### *Plasmodium falciparum* isolates and samples size

A set of 533 blood samples collected in 2010–2011 from *P. falciparum* malaria patients was analysed. These samples originate from 16 health centres (11 health centres and five reference hospitals) located in 10 provinces in Cambodia. Isolates were grouped in four regions: western, southern, eastern and northern Cambodia (Additional file 1). Four control DNA samples were used to validate barcode detection (3D7, Dd2, HB3 and RO33).

### DNA extraction and PCR amplification

The genomic DNA was extracted from 200 µl of blood using the DNA Mini blood kit (Qiagen, Germany) according to manufacturer’s instructions. DNA extracts were stored at −20 °C until use. DNA from reference strains 3D7, Dd2, HB3 and RO33, provided by Malaria Research and Reference Reagent Resource Center-MR4, were used as controls. Primary PCR was carried out in 25 µL of final volume with 5 µL of DNA, 0.25 µM of each corresponding primers (Additional file 2), 0.2 mM of each deoxynucleoside triphosphate (dNTP) (Solis Biodyne), 1× of reaction Buffer, 2.5 mM of MgCl2, 1.25 U FirePol^®^ Taq DNA Polymerase (Solis Biodyne), with the following conditions: 94 °C for 15 min, then 30 cycles of a three step program (94 °C for 30 s, 52–55 °C for 1 min and 72 °C for 1 min) and final extension at 72 °C for 10 min to reach the corresponding target between 164 and 385 bp (Additional file 3).

The nested PCR was performed in 25 μl containing 0.5 μM of each primer (Additional file 2), 0.2 mM of each deoxynucleoside triphosphate (dNTP) (Solis Biodyne), 1× of reaction Buffer, 2.5 mM of MgCl_2_, 2.5 U Taq polymerase (FirePol^®^ DNA Polymerase, Solis Biodyne). 5 µl of the primary PCR reaction were used as the template. PCR conditions were: 94 °C for 15 min, then 40 cycles of 94 °C for 30 s, annealing temperature between 55 and 60 °C for 1 min and 72 ^°^C for 1 min. A final extension at 72 °C for 10 min was performed to obtain the corresponding fragments between 100 and 200 bp. PCR of valid SNPs were performed in four multiplexed reactions (Additional file 3).

### Ligation and detection assays

Nested PCR products were pooled together in two sets according to microsphere combinations. One microlitre of the pooled PCR products were used for the ligase detection reaction (LDR). The LDR was based on two allele-specific primers and one locus-specific probes (Additional file 4). The allele-specific primers were composed of two parts: the 5-prime part hybridizing with the MagPlex-Tag probe and the 3-primer part hybridizing with the PCR product. 33 different MagPlex-Tags were used to detect 40 alleles (Additional file 4) corresponding to the 20 loci which were successfully amplified by PCR (Additional file 3). Ligation was performed after hybridization of the locus-specific primer. Several MagPlex anti-Tag probes were used twice. Locus-specific probes were 5′ phosphorylated and 3′ biotinylated. LDRs were performed in a final volume of 15 µL holding in 1× of Taq Ligase buffer, 10 nM of each LDR (allele- and locus-specific primers), 4 U of Taq DNA ligase (Genesearch) and 1 µL of pooled Nested PCR. Thermocycling conditions were carried out by denaturation of the double stranded DNA at 95 °C for 1 min, followed by 32 cycles at 95 °C for 15 s and hybridization at 58.0–60 °C (Additional file 3) for 2 min. Quality control was performed by using DNA from reference strains provided by MR4. Two multiplexed reactions were used to characterize final valid SNPs (Additional file 3).

### Hybridization and labeling of magnetic beads

A 5 µL fraction of the LDR product was poured into 60 µL of hybridization solution TMAC buffer (3× of tetramethylammonium chloride [TMAC] (Sigma-Aldrich), 3 mM of EDTA (Gibco), 50 mM Tris–HCl, pH 8.0 (Sigma-Aldrich), 0.1 % sodium dodecyl sulfate) and 1000 beads of each MagPlex-Tag microspheres used in the multiplex LDR, as described above. Beads quantification was performed as previously described [13]. Mixtures were heated to 95 °C for 1 min 30 s and incubated at 37 °C for 35 min to allow hybridization between SNPs-specific LDR products (Tag-probe) and bead-labeled anti-TAG probes. Then, 6 µL of 1:50 dilution of streptavidin-R-phycoerythrin (Invitrogen) in TMAC buffer was added to the post-LDR mixture and incubated at 37 °C for 20 min in 96-well plate (Eppendorf). PCR and LDR reactions were conducted in 96-well plate. The fluorescence of each allele-specific LDR products was measured on a MagPix instrument with xPonent 4.2 software (LUMINEX).

The measurement of the signal for an allele was decomposed into the signal intensity without noise and the background noise. Negative samples show reduced signal-to-noise ratio and positive samples show increased signal-to-noise ratio. The identification of negative and positive samples was based on a classification method which minimizes the variance associated to the two series of measures. This algorithm was analogous to the k-mean algorithm where k = 2. A test was used to address each measurement to the negative or positive value of the allele. Negative and positive results for the two alleles were combined to assess barcode value at considered position. Double negative results were considered as positive for the third allele at BC07 barcode position (BC07_ALT_G allele). DNA of the four reference strains 3D7, Dd2, HB3 and RO33 was introduced as the positive control on each 96-well plates. A set of 282 samples were successfully genotyped out of the 533 blood samples initially selected for the analysis, using an 11 positions barcode.

### Data and statistical analysis

Comparison with the available genomic resources was performed after calling of the mutations from BAM files deposited in the ENA database for 167 samples isolated between 2007 and 2011. Correspondence of these isolates and the earlier defined subpopulations in Cambodia [8] was provided by O. Miotto. Alleles for the 11 barcode positions and *k13* locus were recovered from the VCF files.

**Fig. 1:**
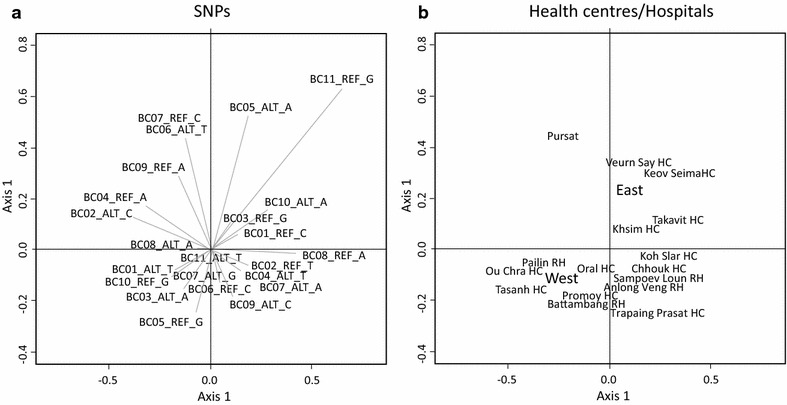
Relationship between allele distribution and geographic origin of parasites in the *P. falciparum* Cambodian population. Correspondence analysis was based on 23 alleles and was conducted for 282 samples. Each reference (REF) and altered (ALT) alleles are represented. Position BC07 had two alternative alleles. **a** Contribution of each allele in the distribution of the 282 samples. **b** Between-class analysis performed with health centres. Analysis was done on the same dataset as in **a**. Scales of the two diagrams are identical

Statistical analysis was performed on the 282 samples using R software [14]. For determining the dependency of the alleles of some selected genes on the locations, firstly the association between alleles and health centres was visually addressed using correspondence analysis (Fig. 1). Analysis was performed for the 11 barcode positions. Space distribution of health centres was questioned using Between-Class analysis. The significance of differences in allele distribution was tested using Chi squared tests for independence. Loci presenting a *p* value <0.05 were considered as exhibiting a significantly different distribution among centres. To identify localities that account most for the SNP allele dependency on health centres, a threshold value of 1 for the Chi squared test statistics components was used (one component for each health centre, Additional file 1). Weblogos [15] were used to highlight conserved alleles among health centres.

Average F_ST_ (fixation index) was calculated to measure the extent of genetic differentiation within health centres. It was based on 11-SNPs barcode of 282 samples. An average allele diversity value was first calculated per sampling area (Hs) from the 11-SNPs barcode. It was then compared with the allele diversity measured for the 282 samples (Ht) to obtain the average F_ST_ value, ranging from 0 (no differentiation/high diversity) to 1 (complete differentiation/low diversity: subpopulations fixed for different alleles).

To define the population structure, hierarchical clustering was performed on 282 samples described by the 11-SNPs barcode. The pairwise distances between the samples were estimated as the proportion of base substitutions between them over the barcode. Ward’s minimum variance method was used to build the dendrogram (Additional file 5A). Random sampling was performed in order to obtain robust results: the clustering approach was implemented on 10,000 subsets of 230 samples each, randomly selected out of the 282 samples. Based on these 10,000 clustering results, pairwise distances between samples were calculated as the percentage of clustering results in which two samples are in the same cluster. This distance matrix was then used to build a dendrogram for all 282 samples. The number k of final clusters (conserved groups) was selected based on the dendrogram structure (Additional file 5B). The value for k = 9, 10 and 14 were producing relevant clusters, but on comparing the barcodes with the previously defined population structure [8], it was observed that the clusters produced on increasing k after 9 are not associated to a specific resistant population (KH2, KH3 and KH4) and instead are related to admixed population KHA and the mixture of other resistant subpopulations. These unspecific clusters were localized close to centre of the country. Hence, the population structure was represented by nine conserved groups (G1 to G9). Weblogo was used to highlight conserved alleles among health centres in G1 to G9 groups. The groups are geolocalized by the geographical centroids on the 2D map. The coordinates for the geographical centroids are calculated as the average of the coordinates (measured in the coordinate space of the 2D map) of the health centres included in the group. To determine the groups significantly related to geographical locations, the average distance to geographical centroid within a group was compared to the average distance to the geographical centroid when the 282 samples were randomly assigned to the nine groups. The average distance for each group was compared to the average distances calculated for 5000 random re-sampling of the 282 samples within the nine conserved groups. The *p* value was calculated as the proportion of average values below the average distance of the health centres to the geographical centroid for each group.

## Results

### Barcode of alleles from 11 variable sites

PCR fragments were successfully amplified for 20 SNPs. There were no PCR amplification for the other four SNPs and were rejected. Assays for only 13 SNPs provide interpretable LUMINEX signal (Additional file 3) and others were excluded from the assay. Out of the 13 SNPs, the locus #7 was abandoned due to non-reproducibility and non-accuracy of the detection on control DNAs and locus #5 was rejected because genotyping analysis revealed that this locus was monomorphic. Finally, 11 SNPs were validated for barcoding (BC01 to BC11). Four multiplex PCRs and two multiplex LDRs were set up for the LUMINEX detection according to their annealing temperature.

Since the work of Daniels et al. [11], the genome version and annotation has been improved. Databases revealed that 23 SNPs amongst the 24 were located in a coding region and they are equally distributed between synonymous and non-synonymous mutations (Additional file 6). Five SNPs were located in subtelomeric regions. Genomic analysis revealed that non detection in the locus #11 was due to the presence of two nearly identical copies of the *rifin* gene in which the SNP is located (Additional file 6). PlasmoDB v11.1 suggested that locus #15 corresponding locus was tri-allelic. LUMINEX data treatment was adapted for this locus. Locus #24 (BC11) was validated for LUMINEX genotyping despite its low variation in Cambodian parasite population (Additional file 6). Initial analysis was performed on 533 samples, 79 were resulting from mixed infection, 183 of the samples present no significant signal with LUMINEX for at least one barcode position and 50 samples could not be amplified using PCR for at least one locus. Among the 251 rejected samples, 59 samples show more than one type of errors (Additional file 7). Finally, 282 samples among 533 blood samples were successfully genotyped at 11 SNP loci.

### Allele distribution associated to health centres

Correspondence analysis was performed using barcode of all the samples (Fig. 1a). Axis 1, 2 and 3 were explaining 21, 16 and 12 % of the information respectively. For each SNP, REF (reference 3D7) and ALT (non-reference) alleles were in opposite quadrants except for BC07 barcode position which was tri-allelic. The BC11_ALT_T allele was located at the centre of the representation as it was present in nearly all samples. Despite the low number of isolates with corresponding BC11_REF_G allele, correspondence analysis showed association of this allele with eastern and southern Cambodia. Association of alleles with health centres was questioned using Between-Class analysis (Fig. 1b). Matching of relative position of health centres in the Between-Class analysis with their geographic position suggests that some alleles show association with samples geographic origin. A strict opposition between eastern and western Cambodia was observed, which can be due to specific distribution of BC03 and BC05 alleles in samples from these areas (Fig. 1). Northern and southern localities present a similar distribution pattern in other projections (Additional file 8). Present analysis suggests that allele frequencies are in agreement with the geographic location of health centres. Pursat is not in a correct position in both correspondence and Between-class analysis. It is located in the western part of Cambodia and it clusters with eastern localities. The discrepancy with its geographic localization could be due to the BC06_ALT_T and BC07 allele frequencies.

### Allele frequency gradient between localities

**Fig. 2:**
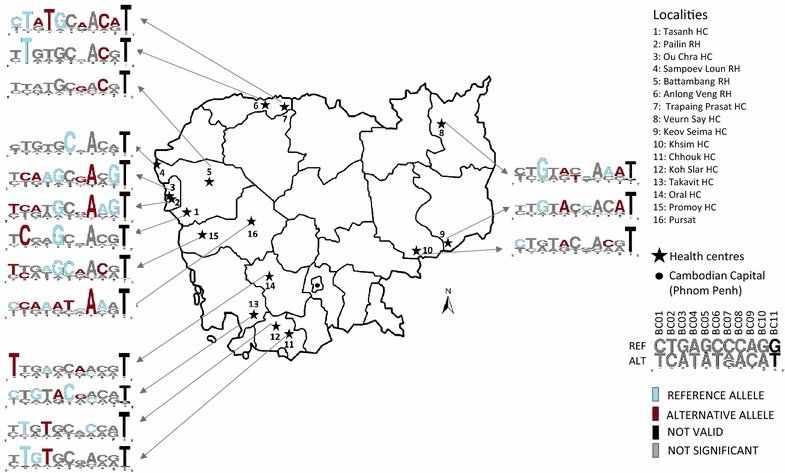
Uneven distribution of alleles in the *P. falciparum* population over Cambodia. *Barcode* was determined per sampling areas. The *barcode* is represented by 11 genomic positions presenting two types of allele per site: the reference allele which is found in 3D7 reference genome (REF) and the alternative allele (ALT). Difference in frequency of one of the REF/ALT allele in a local parasite population vs the 282 samples was evaluated using a Chi squared analysis. The allele was in* blue* for REF and* dark red* for ALT. The allele was in* grey color* when the Chi squared parameter measuring the difference between observed and calculated value was below 1. *Barcode* position BC11 was not suitable for Chi squared analysis

Uneven distribution of alleles was confirmed by Chi squared analysis (*p* value <0.05) and the allele frequencies were represented using Weblogo (Fig. 2). Barcode BC11 was excluded from this analysis because of its low variation. Most important allele enrichments were highlighted in red and blue colors for ALT and REF alleles respectively, using the Chi squared values (Additional file 1). Allele enrichment was not restricted to a single health centre but is often present in geographically close localities. Western Cambodia, including Battambang, Pailin and Pursat provinces displayed significant enrichment of ALT alleles for BC01, BC02, BC03, BC07 and BC08 and the REF allele for BC04, BC05 and BC06 (Additional file 8). The region of Kampot (Koh Slar and Chhouk health centres) showed strong enrichment of BC02_REF_T and BC04_ALT_T with a quasi-absence of opposite alleles (Additional files 1, 9B, D). For some alleles, the gradient from ALT to REF significant allele frequency was emphasized by the presence of health centres located between ALT and REF significant geographic area where no significant enrichment could be specified for any of the alleles. For example, western Cambodia appeared as the starting point for the diffusion of BC02_ALT_C and northern Cambodia was associated with BC02_REF_T (Additional file 9B). The region of Battambang shows no significant allele frequency for this locus. An example for the West-East axis is observed for BC10 locus where the region of Pailin was associated with BC10_REF_G alleles whereas BC10_ALT_A allele was found in eastern Cambodia. Accordingly, Battambang and Pursat health centres located between these two areas show no significant bias in allele frequency (Additional file 9J). Therefore, association between barcode alleles and localities could suggest the presence of specific subpopulations with fixed allele in restricted geographic distribution and overlap between these subpopulations or even gene flow.

### Presence of fixed alleles at the border of Cambodia

**Fig. 3:**
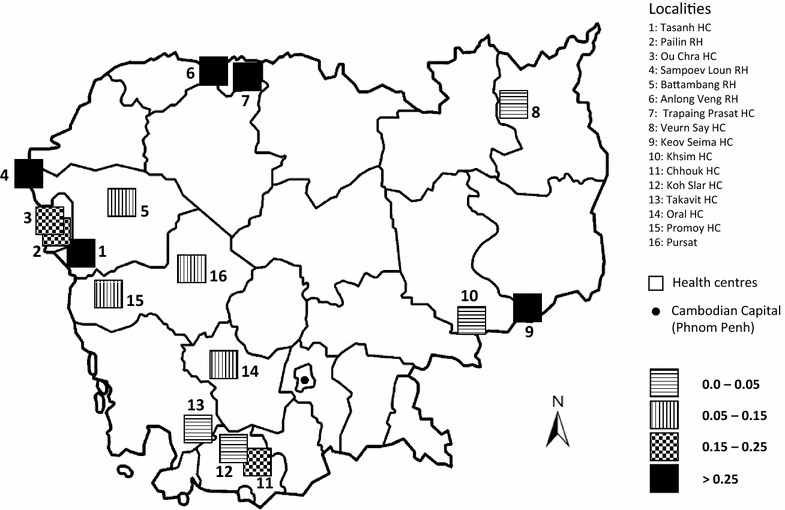
Genetic diversity (Average F_ST_) of the *P. falciparum* population among health centres. The Average F_ST_ value was calculated among all individuals isolated from a specific health centre for the 11 *barcode* positions

The presence of subpopulations was confirmed using an average F_ST_ value calculated per health centres. High F_ST_ values are observed at the localities near the borders of Cambodia (Fig. 3), including Keov Seima (eastern Cambodia). Tasanh and Sampov Loun health centres in western Cambodia are associated with high F_ST_ values and accordingly BC02_ALT_C allele was observed to be fixed in Tasanh region. Similarly, BC04_ALT_T and BC09_ALT_C might have contributed to high F_ST_ values in northern localities. The fixation of BC04_ALT_T allele was also observed in Kampot province (Chhouk HC).

F_ST_ analysis and gradients of allele frequencies (Additional file 9A–J) over the country suggest gene flow in a centripetal orientation. According to high F_ST_ values, the five locations Anlong Veng, Keov Seima, Sampoev Loun, Tasanh and Trapaing Prasat might be associated with parasite subpopulations. Crossing of subpopulations could be responsible for allele diffusion over the country. Especially in western Cambodian sites, where the low F_ST_ values could result from overlap between subpopulations. This likely reflects gene flow driving the homogenization of the population.

### Identification of emerging subpopulations in Cambodia

**Fig. 4:**
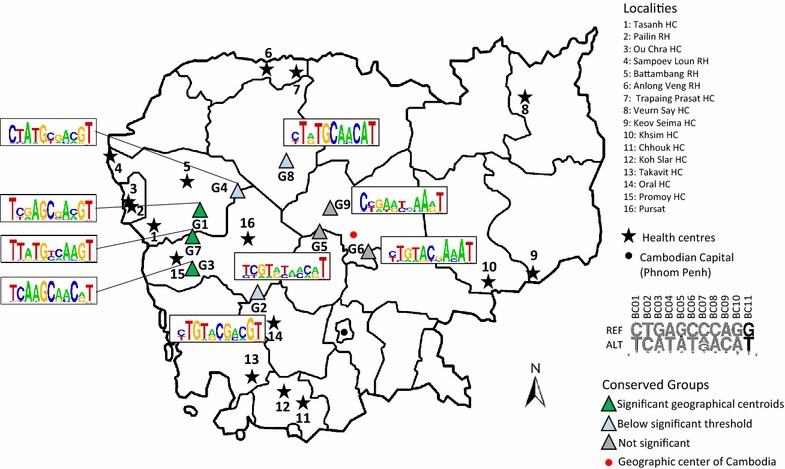
Geographic distribution of the 282 isolates clustered in nine groups and Weblogo representation of the consensus sequence. *Barcodes* were grouped together after 10,000 random pairwise distance comparison of 230 samples. Isolates were grouped together based on the number of time they were clustered together after the 10,000 simulations. The Weblogo provide information on the level of diversity per *barcode* position in each group. Groups were geolocalized using the geographical centroid of samples within the group. The significance of each group was established by comparing the mean distance of the samples to the geographical centroid within a group to the distribution of average distance after random sampling within groups. The *p* value <0.05 was significant

Results presented in sections above suggest that subpopulations were restricted to small geographic areas. Unsupervised clustering runs based on different random subset of the 282 isolates suggested the existence of 9 robust clusters (referred as G1–G9, size of the groups, n = 18–44) representative of the parasite subpopulations. The relationship between groups and health centres was established based on distance of samples to their geographical centroid. None of the groups had samples restricted to a single health centre, and most of the geographical centroids are focused in north-west area of the country (Fig. 4).

The three groups G1, G3 and G7 were significantly associated to specific geographic area (*p* value <0.05). The samples in these groups were mostly isolated in western Cambodia, but also include samples from the north or from the south of the country (Additional file 10). G1 includes two samples from eastern Cambodia. G3 samples were originating from western and southern Cambodia only. G7 had two samples from the southern and one from northern Cambodia. Relationship with previously described Cambodian parasite subpopulations shows that the three groups could be associated with KH2 and KH3 subpopulations. Accordingly, samples that have been probed, carried C580Y *k13* mutation. Weblogos were added to the analysis to illustrate the frequency of alleles at the 11 barcode position among conserved clusters (Fig. 4). In accordance with the results presented above (Figs. 1, 2), G1 shows conserved allele positions: BC01_T, BC04_A, BC05_G, BC08_A and BC10_G. This genotype was very close to the two barcodes associated with Pailin and Ou Chra health centres. Group G3 weblogo was more reminiscent of Promoy HC barcode (Additional file 11). The barcode analysis based on 11 SNPs was efficient to describe conserved subpopulations that emerged recently in western Cambodia concomitantly with artemisinin resistance.

The three groups G2, G4 and G8, are localized in the area between north-western region and the centre of the country (Fig. 4; Additional file 10). The average distance of these groups to the geographical centroid presents a *p* value between 0.1 and 0.3. The two groups, G2 (n = 32) and G4 (n = 27), have samples from various localities. Most of the samples in G2 are originating from localities in southern Cambodia and include 10 samples with C580Y *k13* allele. Three barcodes in this group are found in the admixed KHA subpopulation described earlier [8]. The samples in the group G4 are mostly originating from localities in western Cambodia and include eight samples with C580Y, two samples with R539T and one sample with N458Y *k13* allele. Two barcodes in this group are identified in the previously defined parasite subpopulations, one barcode is found in KH4 subpopulation (also carrying Y493H allele) and the other is found in KHA subpopulation (also carrying R539T allele). The samples in the group G8 (n = 44) are mostly originating from the localities in the north (Trapaing Prasat and Anlong Veng health centre) of the country and include four samples with C580Y and six samples with R539T *k13* allele. In this group the 11 barcode loci are conserved in most of the samples and some samples have variation at BC01, BC03 and BC08 locus. Three barcodes in this group are identified in the previously defined KH3 (shown to carry R539T alleles), KH1 (ancestral population) and KHA (admixed population with C580Y alleles) subpopulations.

The three other groups G5, G6 and G9 are localized close to the centre of the country and show no significant geographical centroid *p* values (0.99, 0.99 and 0.44, respectively). The samples in these groups are originating from localities from all over the country. In the group G5 (n = 40) only four samples are originating from the localities in the south of Cambodia. This group includes four samples with C580Y, one sample with R539T, one sample with P553L and one sample with Y493H *k13* alleles. Four barcodes of this group are identified in the KH subpopulations, two barcodes in KH1 and two barcodes in KHA. The samples in the group G6 (n = 33) are mostly coming from the localities in the southern and eastern regions of Cambodia. This group includes six samples with C580Y, one sample with I543T and one sample with V568G *k13* alleles. Only one barcode of this group is identified in the KH3 subpopulation. The samples in the group G9 (n = 27) are mostly originating from the localities in eastern and western regions and 4/5 tested sample are negative for *k13* allele. In this group, two barcodes are identified in the KH4 subpopulation, two barcodes in the KHA subpopulation, one barcode in KH1 subpopulation and one barcode in KH3 subpopulation. The relationship between barcodes matching the KH4 subpopulation and Y493H allele was confirmed for seven isolates of this group.

The map (Fig. 4) represents a gradient of distribution of relevant subpopulation based on barcode description from north-west to the centre of Cambodia emphasizing gene flow in that orientation. The barcodes of the groups including samples from the north-western localities are mostly associated to the KH2 and KH3 subpopulations and most of the samples carry C580Y *k13* allele only. Moving towards the centre it is observed that the barcodes are associated more with the KHA, KH1 and a specific KH3 subpopulations in the north (G8). The samples are mostly carrying C580Y and R539T *k13* alleles and also a rare N458Y allele in one of the samples. The groups in the centre of the country are including more barcodes associated to the KHA and KH1 subpopulations and some barcodes matching the KH3 and KH4 subpopulations. The samples are shown to carry C580Y, R539T, Y493H and the three rare mutations P553L, I543T and V568G (Additional file 10). This could suggest the localization of the admixed populations with high diversity towards the centre of the country.

### Mefloquine resistance is strongly associated to northern Cambodia

Mutations in *k13* gene associated with resistance to artemisinin were determined in 98 patients, as described earlier [6]. From these patients, 70 % of the samples were positive for one of the *k13* resistant alleles (C580Y, R539T, Y493H, I543T, P553L, V568G & N458Y). Artemisinin resistance was more frequent in western and northern Cambodia (Chi squared test *p* < 0.01). The mutant alleles Y493H, I543T, P553L, V568G, and N458Y were found once in the 282 isolates. The C580Y allele was the most prevalent (54/68 positive patients) and was found to be present in all the conserved groups. Thirty-seven different barcodes were found among these 54 samples. No association was found between the C580Y allele and 11-SNPs barcode. The R539T was the second most frequent allele (9 isolates over 68 positive patients) with six isolates belonging to G8, two to G4 and one from G5. Four isolates were from northern Cambodia, three from western and two from southern Cambodia. Barcodes of these nine samples have BC01_REF_C, BC03_ALT_A, BC04_ALT_T, BC05_REF_G, BC09_ALT_C and BC11_ALT_T in common. All these alleles were significantly associated to Trapaing Prasat health centre (Fig. 2; Additional file 11).

**Fig. 5:**
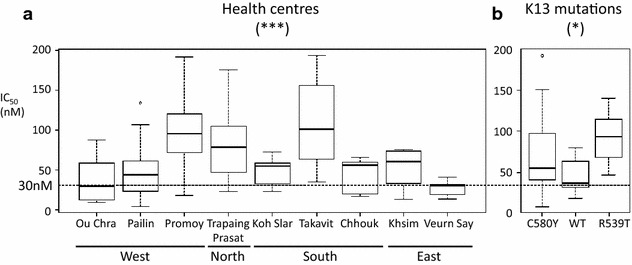
Distribution of mefloquine IC_50_ value among *P. falciparum* isolates in Cambodia. *Box plot* analysis presents median and quartiles. Mefloquine resistant parasite have IC_50_ value over 30 nM (*dashed line*). **a** Mefloquine sensitivity measured per health centres. *** A significant effect was observed (Kruskal–Wallis: *p* = 2.028e−05). **b** Mefloquine sensitivity measured per *k13* gene alleles related to artemisinin resistance. * A significant effect was observed (Kruskal–Wallis: *p* value = 0.01695). Number of samples: C580Y: 29, WT: 15 and R539T: 8

In vitro IC_50_ susceptibilities to chloroquine (n = 109), mefloquine (n = 111), piperaquine (n = 103) and quinine (n = 107), were assessed in isolates with a parasitaemia >0.1 % [16]. Samples were distributed among all geographical locations and clustering groups. Piperaquine showed no geographical bias. The susceptibility for chloroquine and mefloquine were lower in eastern Cambodia (Additional file 12). High mefloquine IC_50_ values were found in isolates from Promoy, Takavit and Trapaing Prasat health centres (Fig. 5). Mefloquine resistant parasites in the region between Promoy and Takavit were mostly carrying C580Y allele. R539T mutant parasites had significantly high mefloquine IC_50_ values (Fig. 5) suggesting two geographic loci for mefloquine resistance in Cambodia, one associated to C580Y allele and one associated to R539T allele. Large proportion of G8 samples were carrying R539T alleles and most originating from Trapaing Prasat HC in the north. The samples in G4 and G8 groups show high mefloquine IC_50_ values (Additional file 13). The F_ST_ values are shown to be high for Trapaing Prasat HC (Fig. 2). These results suggest the presence of a recently emerging *P. falciparum* subpopulation in northern Cambodia.

## Discussion

### Implementation of 11-SNPs barcode for mid-throughput analysis

A PCR-LDR-FMA technique for the Mid-throughput detection of a barcode for *P. falciparum* Cambodian isolates was optimized. This strategy included multiplex PCR and ligase detection reactions prior to hybridization with magnetic microspheres (MagPlex-Tag probes). A specific algorithm was developed for signal discrimination between the 23 alleles corresponding to 11 SNPs which enabled us to characterize 282 samples. The choice of the loci was a critical step. It was based on both possibility to run detection at the corresponding locus and allele frequency. In fact, the PCR-LDR-FMA required the design of several primers at each locus. This was the main source of rejection of SNPs. Indeed, this task was hampered by the AT richness of *P. falciparum* genome. The frequency of alleles in the present dataset (Additional file 6) was sometimes different from the expected non-reference allele frequency (NRAF). One explanation could be that the study of Daniels et al. included only Thai samples [11]. Discrepancy was also observed with the MalariaGEN database. Genome annotation of *P. falciparum* is available at PlasmoDB. Bioanalysis revealed that some of the selected SNPs were present in genes located in subtelomeric regions and encoded surface antigens (Additional file 6). These regions are known to be highly variable and to encode surface antigens. Variation in these regions might not reflect population evolution, but more parasite-host interactions such as antigenic variation.

### 11-SNPs barcode successfully identifies parasite subpopulations

The 11-SNPs barcode analysis at a country-wide scale confirmed that *P. falciparum* population is fragmented into subpopulations. Evidences were given both by individual SNPs and by global barcode analysis. The present study covers all regions where *P. falciparum* is endemic in Cambodia. The uneven distribution of most alleles confirmed the presence of subpopulations with restricted geographic distribution, leading us to define nine groups. Isolates from clustering groups G1, G3 and G7 were mostly located in western Cambodia. They were associated with the C580Y *k13* mutant allele and correspond to the KH2 and KH3 subpopulations described earlier [6, 8]. These three groups might have restricted geographic origin as barcode allele frequencies from health centres (Fig. 2) and clustering groups (Fig. 4) were similar (Additional file 11). Subpopulation in these localities have been shown to emerge recently and correlates with artemisinin resistance specific to C580Y allele. It should be interesting to compare the barcodes in the two subpopulations (WKH-F01 and WKH-F03) described recently in the same localities in western Cambodia [9] from parasites isolated later than the present study (2011–2013).

Among the three less significant groups defined by the barcodes, G2 and G4 could not be associated to any of the specific subpopulations described earlier. The reason for G2 could be the origin of samples from the area which was not covered by genomic analysis. In fact, high F_ST_ value was observed in Chhouk suggesting possible emergence of subpopulation in southern Cambodia. Only five barcodes from these two groups were matching with the genotypes of defined subpopulation origin, one from KH4 and 4 from the admixed subpopulation KHA. The group G8 was associated with northern Cambodia and most of the samples were carrying the R539T *k13* allele. Allele frequencies of this group were very close to that of the Trapaing Prasat HC, as illustrated by the Weblogos (Additional file 11). The high F_ST_ value of northern localities is in agreement with the hypothesis of gene flow associated with R539T mutation from the north. The prevalence of R539T *k13* allele associated with increased ring stage survival and delayed parasite clearance rates near eastern Thailand and northern Cambodia border has been shown in recent studies [17, 18].

The 11-SNPs barcode questions the origin of subpopulations in Cambodia. Subpopulations are expected to emerge at different places and at different times over the country. Introduction of ACT is clearly associated with emergence of western parasite subpopulations. The origin of other subpopulations is not known. Barcode analysis shows that the genetic drift induced by the emergence of subpopulation disappears rapidly over time, most likely through the presence of admixed populations. Unfortunately, the present 11-SNPs barcode seems less relevant for the characterization of populations with high heterogeneity such as core population KH1 and admixed population KHA.

### Relationship between subpopulations and drug susceptibilities

Groups G1, G3 and G7 were associated with resistance to artemisinin. Resistant parasites in these groups were carrying the C580Y allele only (Additional file 10). The diffusion of the C580Y allele in 2010–2011 over Cambodia was described previously [6]. The present study provides evidence that the C580Y allele can be found in all groups. In the group G8, though the C580Y allele is present, most of the samples are associated to the R539T resistant allele. Three groups contains more than one *k13* allele: G4 (N458Y, 1; R539T, 2; C580Y, 8), G5 (Y493H, 1; P553L, 1; R539T, 1; C580Y, 4), G6 (I543T, 1; V568G, 1; C580Y, 6) and G8 (R539T, 6; C580Y, 4). The R539T allele was strongly associated to a subpopulation originating from northern Cambodia and represented by G8 group. The two groups G4 and G8 were associated to high mefloquine IC_50_ values (Additional file 13). Interestingly, G8 includes highest number of samples (21/28) with associated to high mefloquine IC_50_ values (IC_50_ > 30 nM). Parasites were genetically close according to their barcode. Allele frequency in G8 was close from those found in Trapaing Prasat samples, suggesting that it is the region of origin of these parasites. This result is in agreement with the study by Chaorattanakawee et al., which shows the increase in occurrence of R539T allele from 2009 to 2013 and association with increased mefloquine IC_50_ value for R539T allele (ex vivo drug susceptibility test) [19]. Despite the observed association between drug resistance (artemisinin and mefloquine) and subpopulations, the present study provides no evidence that drug pressure is responsible for emergence of subpopulations in western and northern Cambodia.

### Genetic exchange between parasite subpopulations

Clustering approach provided evidence that subpopulations have emerged in different parts of the country. They might have emerged from the ancestral KH1 population described by Miotto et al. [8]. Significance of geographical centroids shows that the most recently emerged subpopulations could be well localized. The decrease of the significance could be related to the diffusion of alleles, which in that case follows the west to east major axis (Fig. 4). Nevertheless, other gene flow axis might also be present over Cambodia. This was confirmed by high F_ST_ values at the periphery of the country (Fig. 3). Regions with high F_ST_ were strongly related to forest areas which are mostly distributed at the border of Cambodia. Parasite subpopulations might have encountered independent drift of mutation and selection. Parasites moving out of their region of origin will progressively mix their genetic background with other parasites. This hypothesis is supported by the presence of G2, G4 and G8 groups containing barcodes associated to admixed population KHA. Genetic exchange between subpopulations takes place by mating. It might be supported by human socio-economical migrations. The presence of this ongoing gene flow might have supported the eastward dispersal of artemisinin resistance *k13* alleles after introducing ACT in the country. Currently, environmental factors such as deforestation, development of communication axes and global welfare are changing rapidly. It will be interesting to develop a specific barcode analysis to follow the evolution of these subpopulations in this new socio-economical context.

## Additional files

10.1186/s12936-016-1370-y Geographic distribution of samples and allele frequency. Blood samples from *P. falciparum* positive patients were collected from 16 health centres or hospitals covering the areas where parasite transmission is active. The number of valid samples (282) out of the total selected samples (533) is provided per health centre. The frequency of each allele (REF/ALT) is given for the 11 loci, which were positive for LUMINEX detection. The alleles with Chi squared test statistics components (one component for each health centre) greater than 1 are highlighted in grey. The presence of Kelch-propeller domain altered allele was assessed by PCR and sequencing. The frequencies of wild type individuals and of the two major alleles C580Y and R539T are provided per location. The other alleles present at low frequency, N458Y, Y493H, I543T, P553L and V568G are pooled together. The drug sensitivity was measured routinely for patients presenting high parasitaemia (>2 %). The number of samples tested for IC_50_ measurements for chloroquine (CQ), piperaquine (PIP), quinine (QN), artesunate (ART), mefloquine (MF) and dihydroartemisinin (DHA) is provided.
10.1186/s12936-016-1370-y Primers sequences for PCR reactions corresponding to 20 of the 24 SNPs selected for barcode detection and for *k13* locus amplification.
10.1186/s12936-016-1370-y PCR and LDR conditions. NV for not valid PCR. Eight LUMINEX assays were negative. Assay#7 was rejected because one allele only was not detected (Pos/Neg). LUMINEX detection was performed after microsphere hybridization and ligation reaction. ID of microspheres that were used for several assays are in bold.
10.1186/s12936-016-1370-y Primer sequences for LDR (Ligation Detection reaction) performed.
10.1186/s12936-016-1370-y Classification of samples into 9 conserved groups. **A**. Hierarchical clustering of the 282 valid samples based on the 11-SNPs barcode. The pairwise distance between the samples is calculated as the proportion of base substitution between them over the barcode. Ward’s minimum variance method was used to build the dendrogram. The dendrogram is cut to obtain 8 clusters (k = 8). The clusters are represented by red rectangles. **B**. Hierarchal clustering of 282 samples based on the percentage of clustering results in which two samples are in the same cluster (when 8 clusters are considered). The clustering approach was implemented on 10,000 subsets of 230 samples each, randomly selected out of the 282 samples. Based on these 10,000 clustering results, pairwise distance between samples were calculated as the percentage of clustering results in which two samples are in the same cluster. The number of clusters (conserved groups) was selected based on the dendrogram structure. The clusters are represented by red rectangles.
10.1186/s12936-016-1370-y Major features of the Single Nucleotide Polymorphisms selected for LUMINEX assay. A set 11 SNPs (highlighted in grey) has been selected from 24 SNPs validated by Daniels et al. [11]. NRAF value from three geographic areas and the global NRAF were recovered from MalariaGEN v4.0. Genome position was evaluated according to genome version 3. Subtelomere were identified based on gene composition. Valid SNPs are highlighted in grey. Frequency of valid alleles was calculated based on the data mentioned in Additional file 3. SNP ID is the column “Position” preceded by the tag “Pf3D7_[01-14]_v3:” in MalariaGEN and by the tag “NGS_SNP.Pf3D7_[01-14]_v3.” in PlasmoDB.
10.1186/s12936-016-1370-y Number of samples rejected due to mixed infection (M), no significant signal with LUMINEX for at least one barcode position (N) and no amplification using PCR for at least one locus (X).
10.1186/s12936-016-1370-y Relationship between allele distribution and geographic origin of parasites in the *P. falciparum* Cambodian population. Correspondence analysis was based on 23 alleles and was conducted for 282 samples. Each reference (REF) and altered (ALT) alleles are represented. Position BC07 had two alternative alleles. Left panel presented the contribution of each allele in the distribution the 282 samples. Between-class analysis performed with health centres is presented in the right panel. **A**. Axis1-axis3 projection of the correspondence analysis. **B**. Axis2-axis3 projection of the correspondence analysis.
10.1186/s12936-016-1370-y Gene flow analysis based on uneven distribution of alleles in the *P. falciparum* population over Cambodia. The barcode is represented by 11 genomic positions presenting two types of allele per site: the reference allele which is found in 3D7 reference genome (REF) and the alternative allele (ALT). Over representation of one of the REF/ALT allele in a local parasite population was evaluated using a Chi squared analysis. The allele was in blue for REF and dark red for ALT. The box was in grey when the Chi squared test statistics components (one component for each health centre) was less than 1. Allele distribution is presented for barcode position BC01 to BC10. Barcode position BC11 was not suitable for Chi squared analysis. Corresponding position in the barcode in surrounded in red. The significant health centres which are close together were circled. **A**. Allele distribution for barcode BC01. **B**. Allele distribution for barcode BC02. **C**. Allele distribution for barcode BC03. **D**. Allele distribution for barcode BC04. **E**. Allele distribution for barcode BC05. **F**. Allele distribution for barcode BC06. **G**. Allele distribution for barcode BC07. **H**. Allele distribution for barcode BC08. **I**. Allele distribution for barcode BC09. **J**. Allele distribution for barcode BC10.
10.1186/s12936-016-1370-y. Sample Meta-information, barcode, associated drug susceptibility, associated *k13* alleles and the correspondence to the KH subpopulations [8] (based on the matching of the barcode). The blank cells in the columns 21-28 and columns 29-35 means that the samples are not tested for drug susceptibility and *k13* alleles, respectively.
10.1186/s12936-016-1370-y Comparison of allele frequencies in health centre and in conserved clustering groups. A comparative analysis was illustrated using Weblogos sorted for health centre and clustering groups.
10.1186/s12936-016-1370-y Distribution of IC_50_ value of *P. falciparum* isolates per major geographic areas. Box Plot analysis is presenting median and quartiles. Dashed line figure out the threshold where parasite could be resistant for the drug (30 nM). Parasites were originating from regions distributed at the four compass points in Cambodia. ANOVA test was significant for chloroquine and mefloquine (*p* value = 5.62e−5 and *p* value = 0.0408, respectively).
10.1186/s12936-016-1370-y Distribution of mefloquine IC_50_ value of isolates associated to conserved clusters G1 to G9. Box Plot analysis is presenting median and quartiles. Mefloquine resistant parasites have IC_50_ over the dashed line (30 nM).
