# Supplementary figures and images for: Plasmodium falciparum parasite population structure and gene flow associated to anti-malarial drugs resistance in Cambodia
Source: Malar J. 2016 Jun 14;15:319. doi: 10.1186/s12936-016-1370-y (PMC4908689; doi:10.1186/s12936-016-1370-y)

## Slide 1
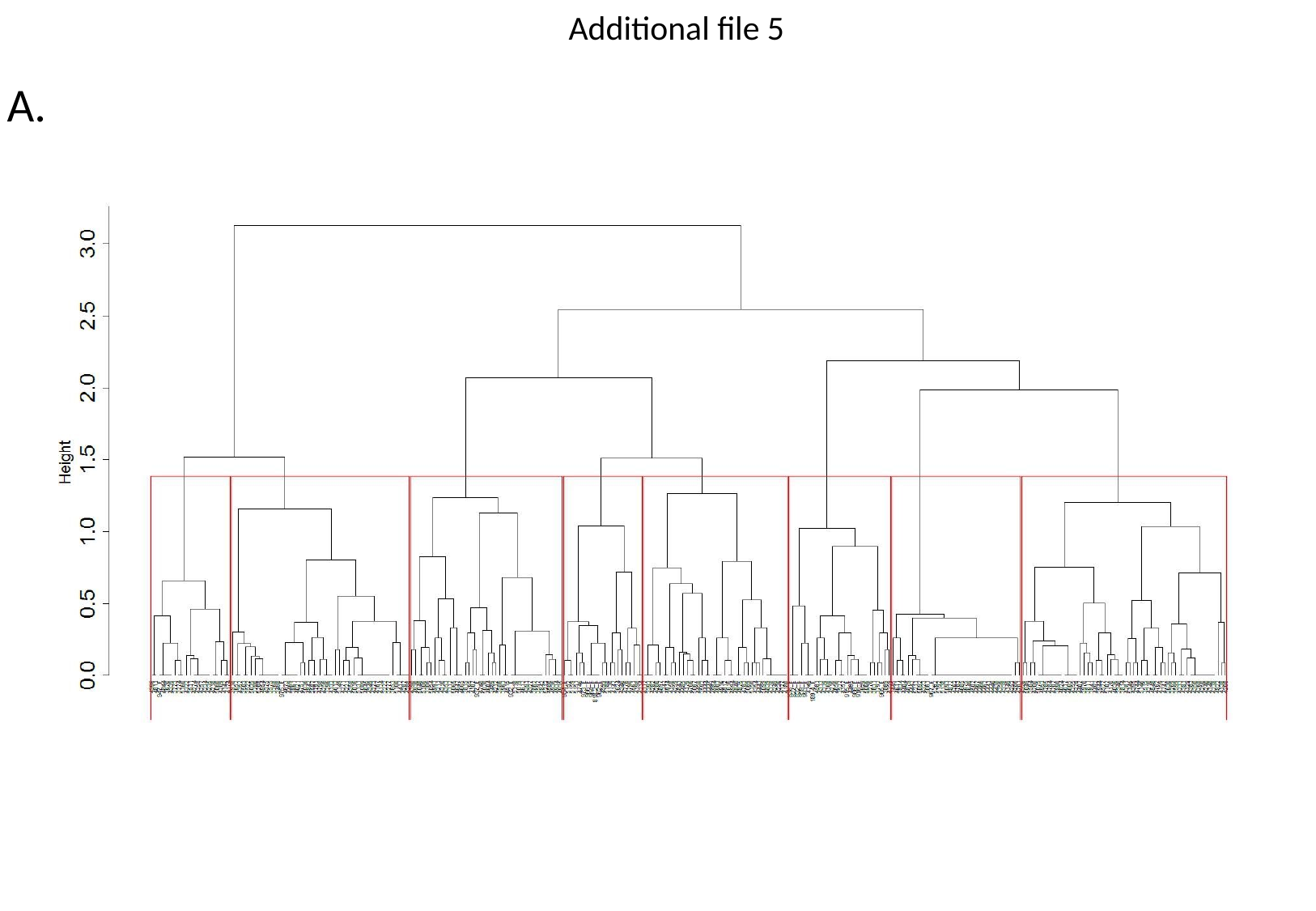

Additional file 5
A.

## Slide 2
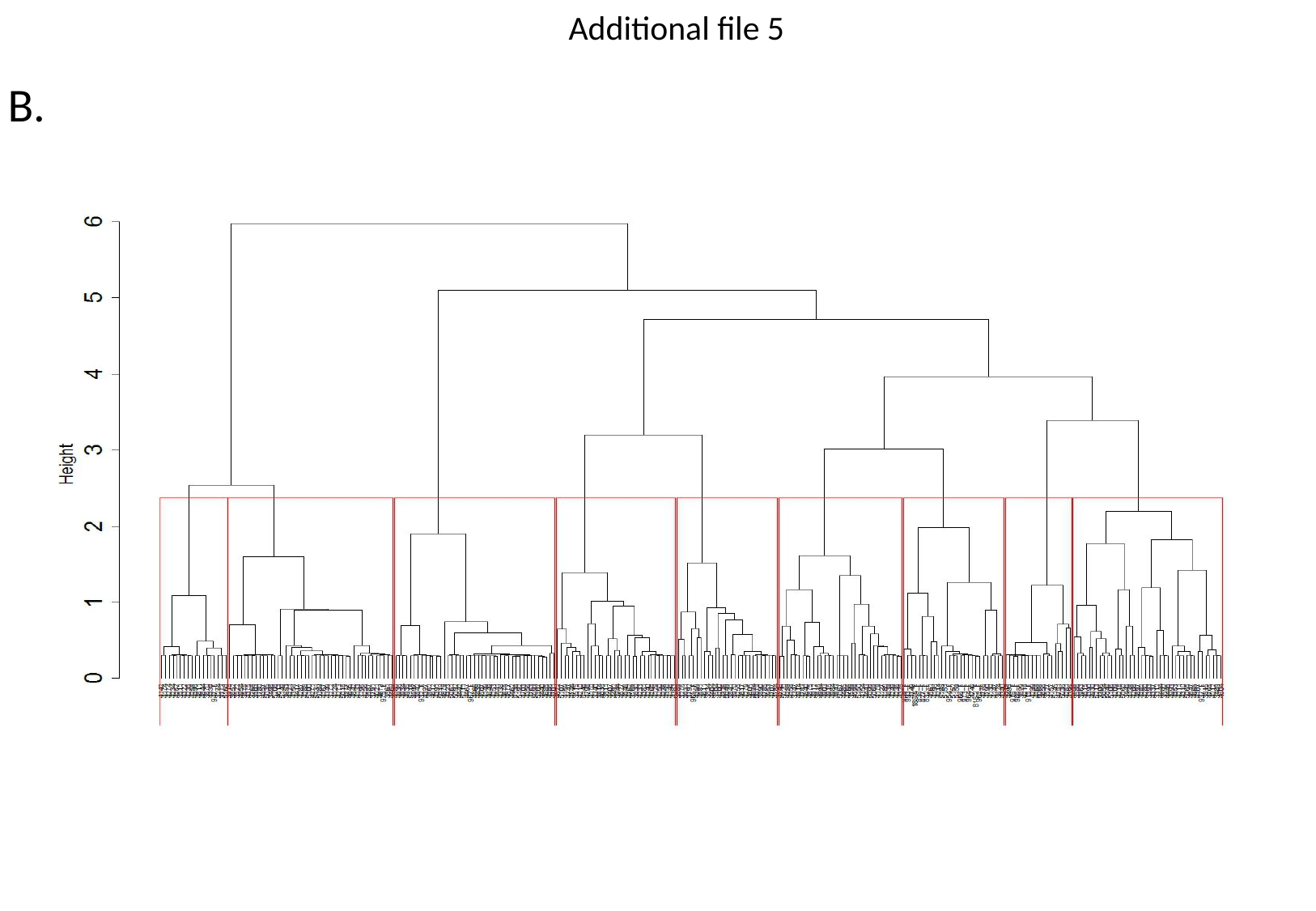

Additional file 5
B.

Supplement: Supplementary file 5 — 10.1186/s12936-016-1370-y Classification of samples into 9 conserved groups. A. Hierarchical clustering of the 282 valid samples based on the 11-SNPs barcode. The pairwise distance between the samples is calculated as the proportion of base substitution between them over the barcode. Ward’s minimum variance method was used to build the dendrogram. The dendrogram is cut to obtain 8 clusters (k = 8). The clusters are represented by red rectangles. B. Hierarchal clustering of 282 samples based on the percentage of clustering results in which two samples are in the same cluster (when 8 clusters are considered). The clustering approach was implemented on 10,000 subsets of 230 samples each, randomly selected out of the 282 samples. Based on these 10,000 clustering results, pairwise distance between samples were calculated as the percentage of clustering results in which two samples are in the same cluster. The number of clusters (conserved groups) was selected based on the dendrogram structure. The clusters are represented by red rectangles. [file 12936_2016_1370_MOESM5_ESM.pptx]

## Slide 1
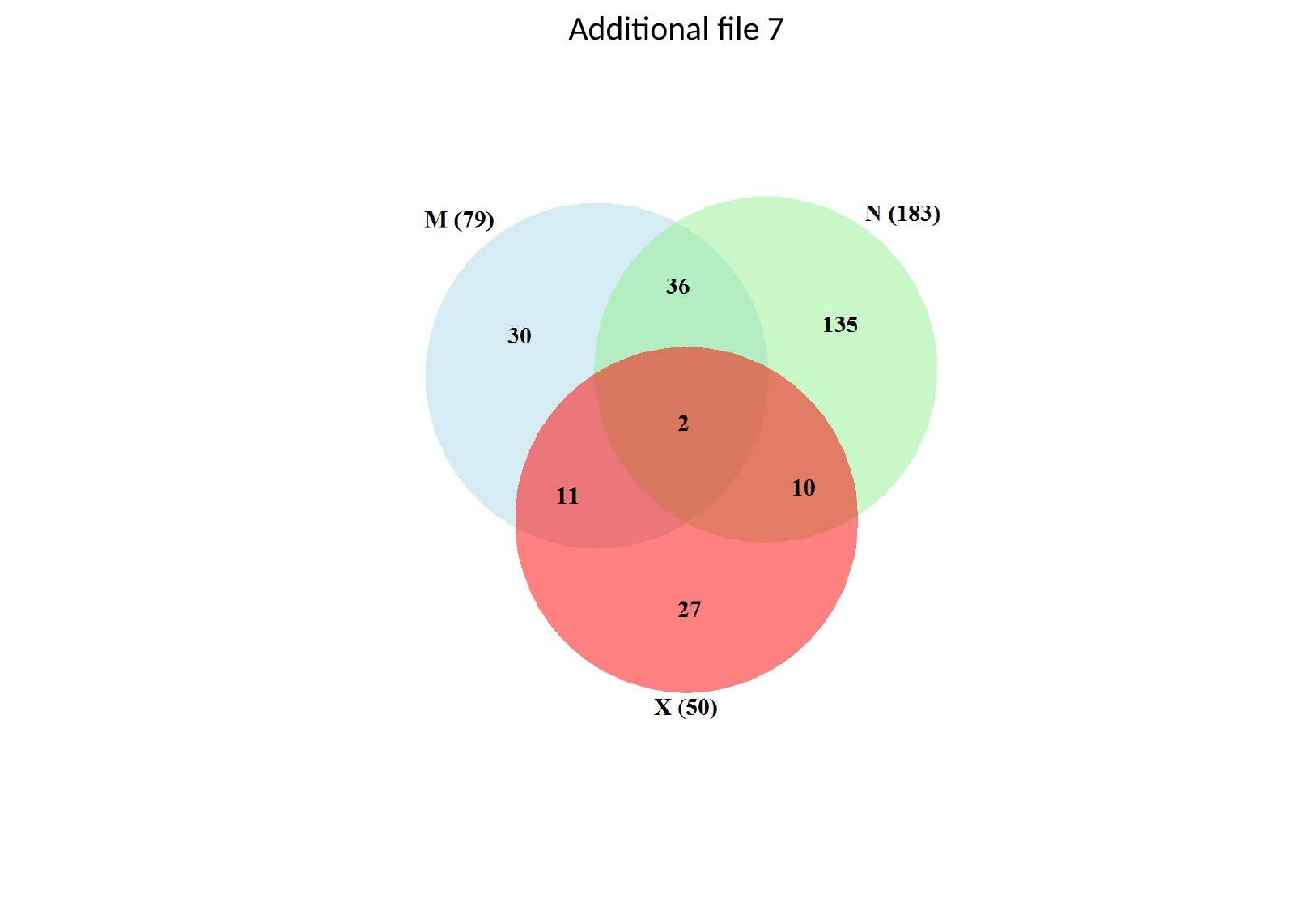

Additional file 7

Supplement: Supplementary file 7 — 10.1186/s12936-016-1370-y Number of samples rejected due to mixed infection (M), no significant signal with LUMINEX for at least one barcode position (N) and no amplification using PCR for at least one locus (X). [file 12936_2016_1370_MOESM7_ESM.pptx]

## Slide 1
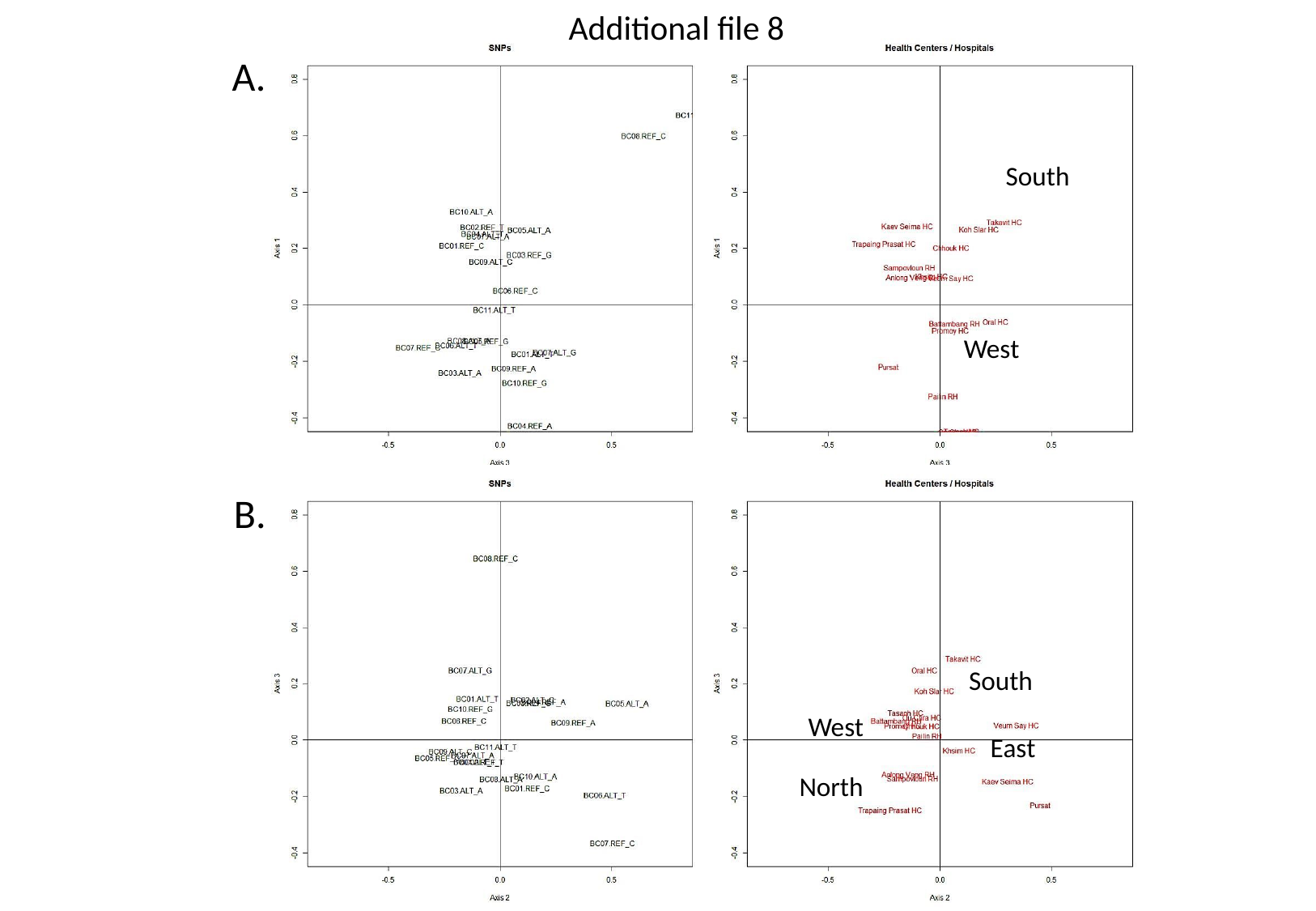

Additional file 8
A.
South
West
B.
South
West
East
North

Supplement: Supplementary file 8 — 10.1186/s12936-016-1370-y Relationship between allele distribution and geographic origin of parasites in the P. falciparum Cambodian population. Correspondence analysis was based on 23 alleles and was conducted for 282 samples. Each reference (REF) and altered (ALT) alleles are represented. Position BC07 had two alternative alleles. Left panel presented the contribution of each allele in the distribution the 282 samples. Between-class analysis performed with health centres is presented in the right panel. A. Axis1-axis3 projection of the correspondence analysis. B. Axis2-axis3 projection of the correspondence analysis. [file 12936_2016_1370_MOESM8_ESM.pptx]
