# Supplementary material for: Plasmodium falciparum parasite population structure and gene flow associated to anti-malarial drugs resistance in Cambodia
Source: Malar J. 2016 Jun 14;15:319. doi: 10.1186/s12936-016-1370-y (PMC4908689; doi:10.1186/s12936-016-1370-y)
Supplement: Supplementary file 9 — 10.1186/s12936-016-1370-y Gene flow analysis based on uneven distribution of alleles in the P. falciparum population over Cambodia. The barcode is represented by 11 genomic positions presenting two types of allele per site: the reference allele which is found in 3D7 reference genome (REF) and the alternative allele (ALT). Over representation of one of the REF/ALT allele in a local parasite population was evaluated using a Chi squared analysis. The allele was in blue for REF and dark red for ALT. The box was in grey when the Chi squared test statistics components (one component for each health centre) was less than 1. Allele distribution is presented for barcode position BC01 to BC10. Barcode position BC11 was not suitable for Chi squared analysis. Corresponding position in the barcode in surrounded in red. The significant health centres which are close together were circled. A. Allele distribution for barcode BC01. B. Allele distribution for barcode BC02. C. Allele distribution for barcode BC03. D. Allele distribution for barcode BC04. E. Allele distribution for barcode BC05. F. Allele distribution for barcode BC06. G. Allele distribution for barcode BC07. H. Allele distribution for barcode BC08. I. Allele distribution for barcode BC09. J. Allele distribution for barcode BC10. [file 12936_2016_1370_MOESM9_ESM.pptx]

## Slide 1
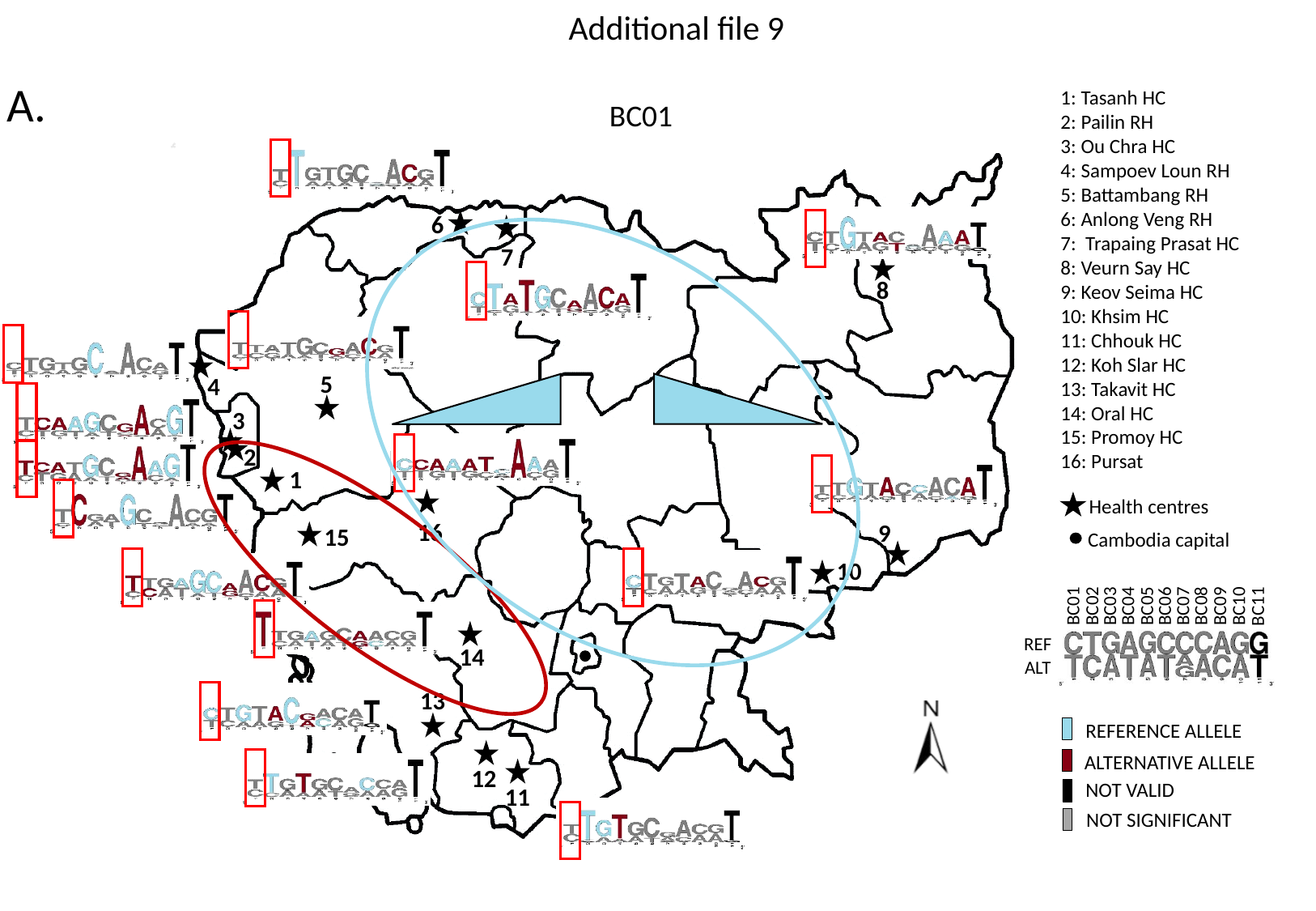

Additional file 9
A.
1: Tasanh HC
2: Pailin RH
3: Ou Chra HC
4: Sampoev Loun RH
5: Battambang RH
6: Anlong Veng RH
7: Trapaing Prasat HC
8: Veurn Say HC
9: Keov Seima HC
10: Khsim HC
11: Chhouk HC
12: Koh Slar HC
13: Takavit HC
14: Oral HC
15: Promoy HC
16: Pursat
BC01
6
7
8
5
4
3
2
1
16
9
15
10
14
13
12
11
Health centres
Cambodia capital
BC01
BC02
BC03
BC04
BC05
BC06
BC07
BC08
BC09
BC10
BC11
REF
ALT
REFERENCE ALLELE
ALTERNATIVE ALLELE
NOT VALID
NOT SIGNIFICANT

## Slide 2
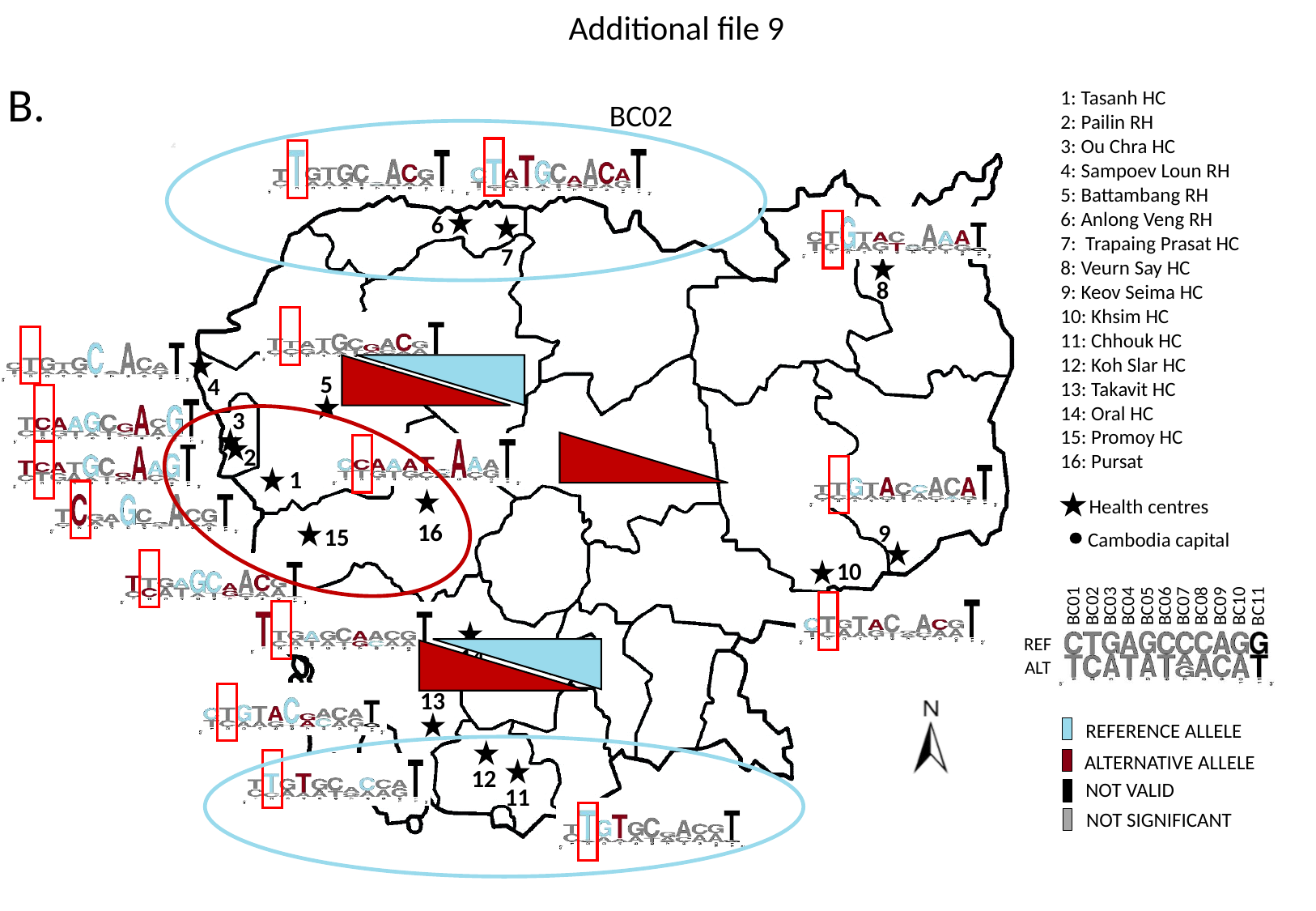

Additional file 9
B.
1: Tasanh HC
2: Pailin RH
3: Ou Chra HC
4: Sampoev Loun RH
5: Battambang RH
6: Anlong Veng RH
7: Trapaing Prasat HC
8: Veurn Say HC
9: Keov Seima HC
10: Khsim HC
11: Chhouk HC
12: Koh Slar HC
13: Takavit HC
14: Oral HC
15: Promoy HC
16: Pursat
BC02
6
7
8
5
4
3
2
1
16
9
15
10
14
13
12
11
Health centres
Cambodia capital
BC01
BC02
BC03
BC04
BC05
BC06
BC07
BC08
BC09
BC10
BC11
REF
ALT
REFERENCE ALLELE
ALTERNATIVE ALLELE
NOT VALID
NOT SIGNIFICANT

## Slide 3
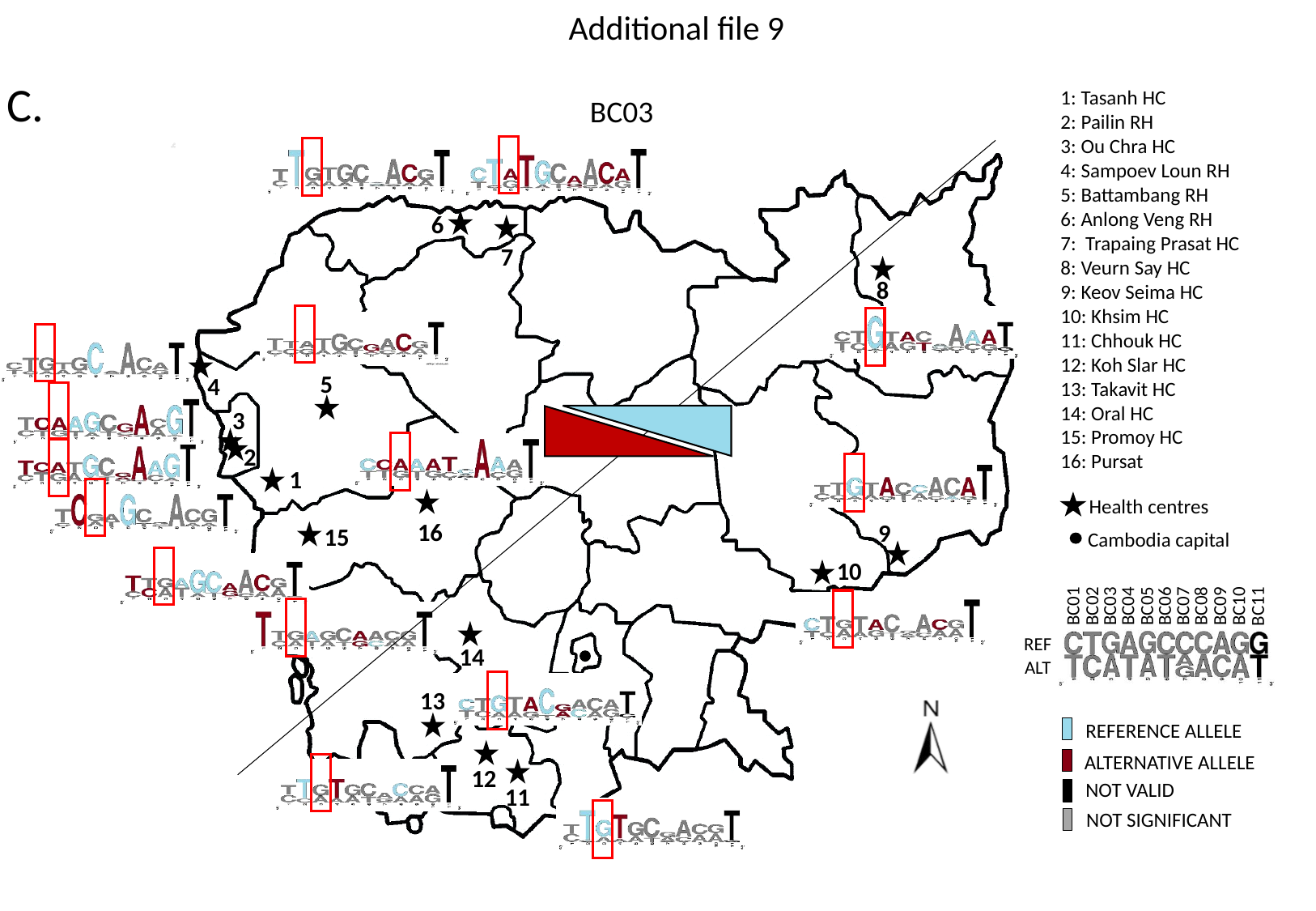

Additional file 9
C.
1: Tasanh HC
2: Pailin RH
3: Ou Chra HC
4: Sampoev Loun RH
5: Battambang RH
6: Anlong Veng RH
7: Trapaing Prasat HC
8: Veurn Say HC
9: Keov Seima HC
10: Khsim HC
11: Chhouk HC
12: Koh Slar HC
13: Takavit HC
14: Oral HC
15: Promoy HC
16: Pursat
BC03
6
7
8
5
4
3
2
1
16
9
15
10
14
13
12
11
Health centres
Cambodia capital
BC01
BC02
BC03
BC04
BC05
BC06
BC07
BC08
BC09
BC10
BC11
REF
ALT
REFERENCE ALLELE
ALTERNATIVE ALLELE
NOT VALID
NOT SIGNIFICANT

## Slide 4
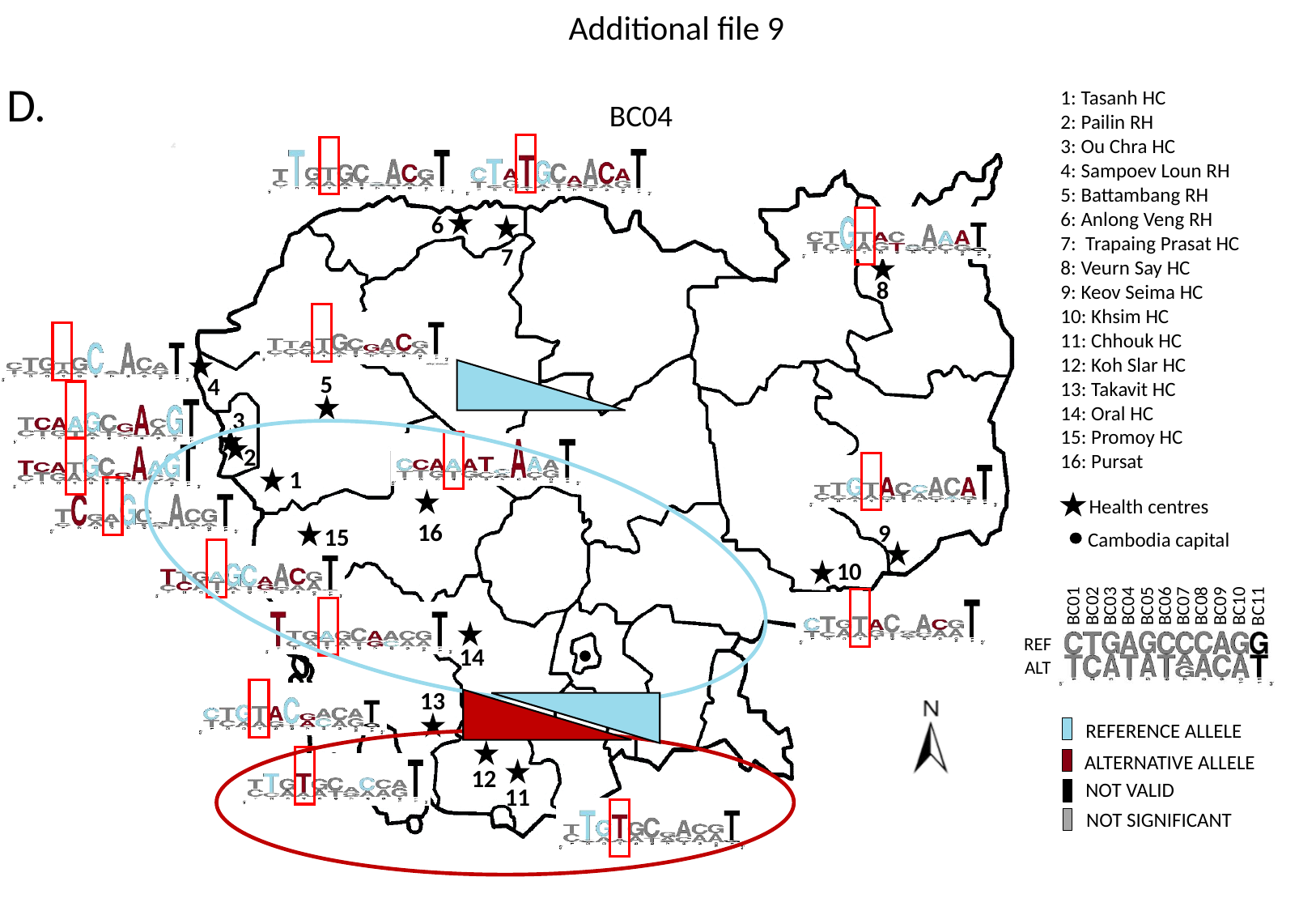

Additional file 9
D.
1: Tasanh HC
2: Pailin RH
3: Ou Chra HC
4: Sampoev Loun RH
5: Battambang RH
6: Anlong Veng RH
7: Trapaing Prasat HC
8: Veurn Say HC
9: Keov Seima HC
10: Khsim HC
11: Chhouk HC
12: Koh Slar HC
13: Takavit HC
14: Oral HC
15: Promoy HC
16: Pursat
BC04
6
7
8
5
4
3
2
1
16
9
15
10
14
13
12
11
Health centres
Cambodia capital
BC01
BC02
BC03
BC04
BC05
BC06
BC07
BC08
BC09
BC10
BC11
REF
ALT
REFERENCE ALLELE
ALTERNATIVE ALLELE
NOT VALID
NOT SIGNIFICANT

## Slide 5
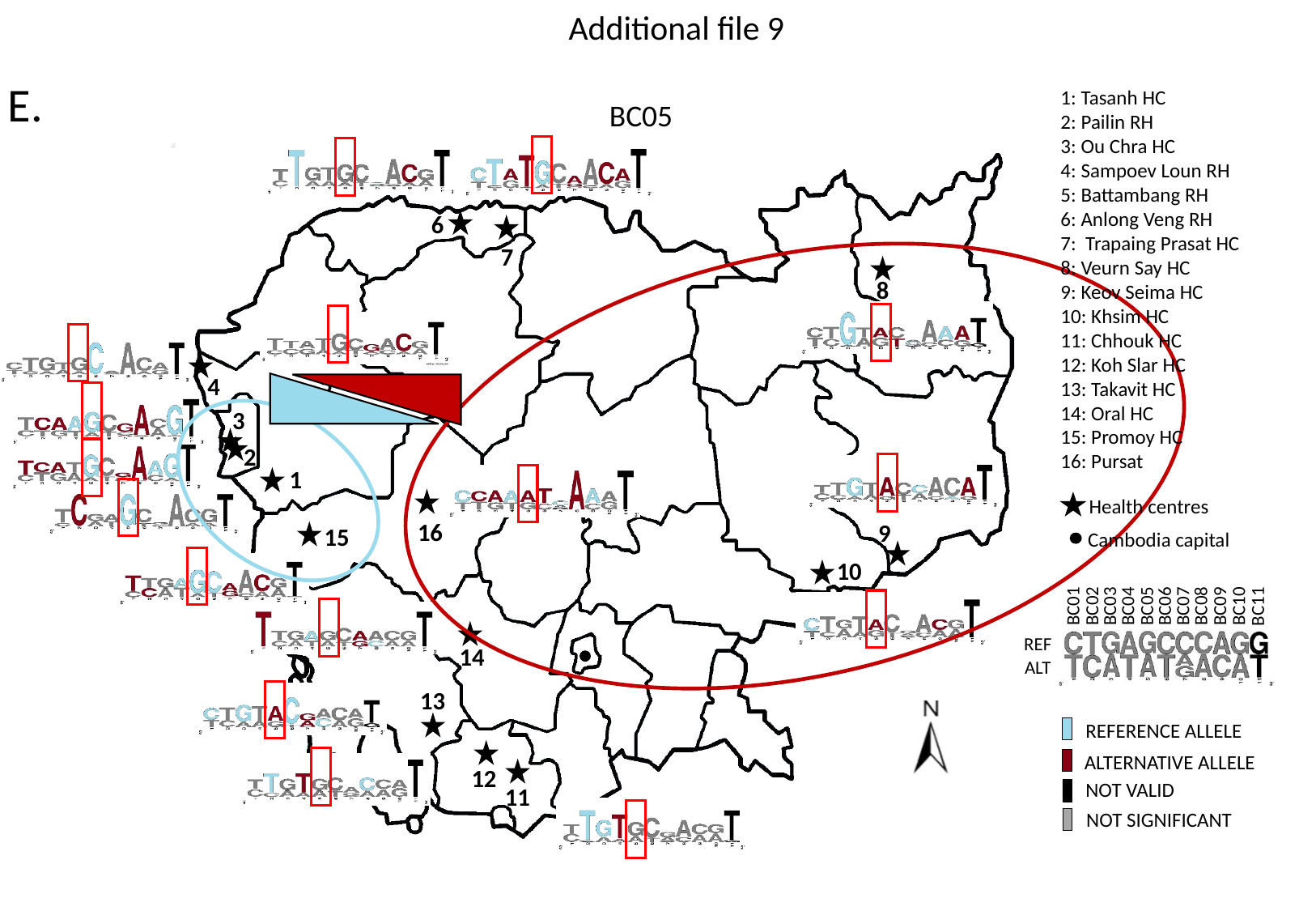

Additional file 9
E.
1: Tasanh HC
2: Pailin RH
3: Ou Chra HC
4: Sampoev Loun RH
5: Battambang RH
6: Anlong Veng RH
7: Trapaing Prasat HC
8: Veurn Say HC
9: Keov Seima HC
10: Khsim HC
11: Chhouk HC
12: Koh Slar HC
13: Takavit HC
14: Oral HC
15: Promoy HC
16: Pursat
BC05
6
7
8
5
4
3
2
1
16
9
15
10
14
13
12
11
Health centres
Cambodia capital
BC01
BC02
BC03
BC04
BC05
BC06
BC07
BC08
BC09
BC10
BC11
REF
ALT
REFERENCE ALLELE
ALTERNATIVE ALLELE
NOT VALID
NOT SIGNIFICANT

## Slide 6
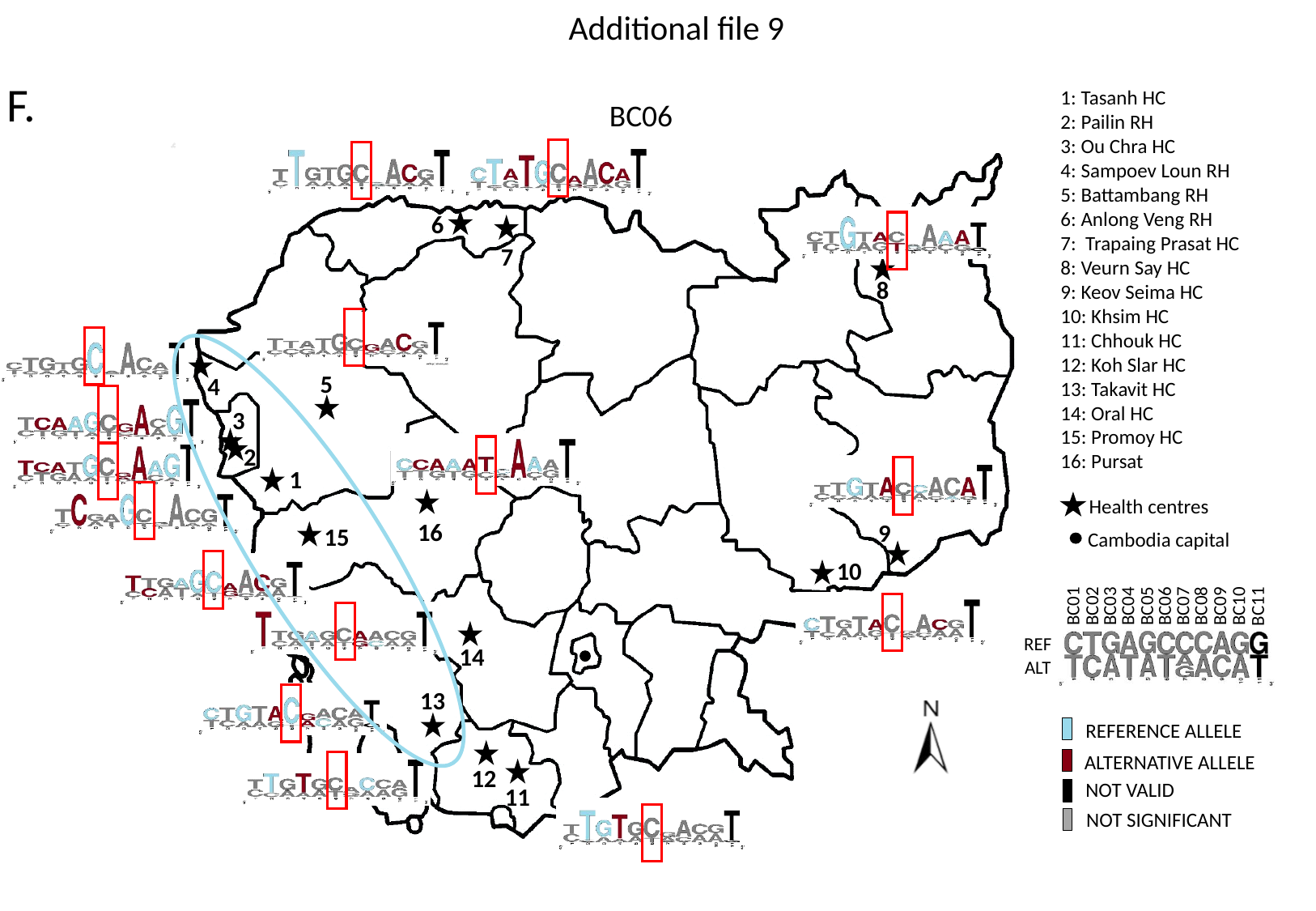

Additional file 9
F.
1: Tasanh HC
2: Pailin RH
3: Ou Chra HC
4: Sampoev Loun RH
5: Battambang RH
6: Anlong Veng RH
7: Trapaing Prasat HC
8: Veurn Say HC
9: Keov Seima HC
10: Khsim HC
11: Chhouk HC
12: Koh Slar HC
13: Takavit HC
14: Oral HC
15: Promoy HC
16: Pursat
BC06
6
7
8
5
4
3
2
1
16
9
15
10
14
13
12
11
Health centres
Cambodia capital
BC01
BC02
BC03
BC04
BC05
BC06
BC07
BC08
BC09
BC10
BC11
REF
ALT
REFERENCE ALLELE
ALTERNATIVE ALLELE
NOT VALID
NOT SIGNIFICANT

## Slide 7
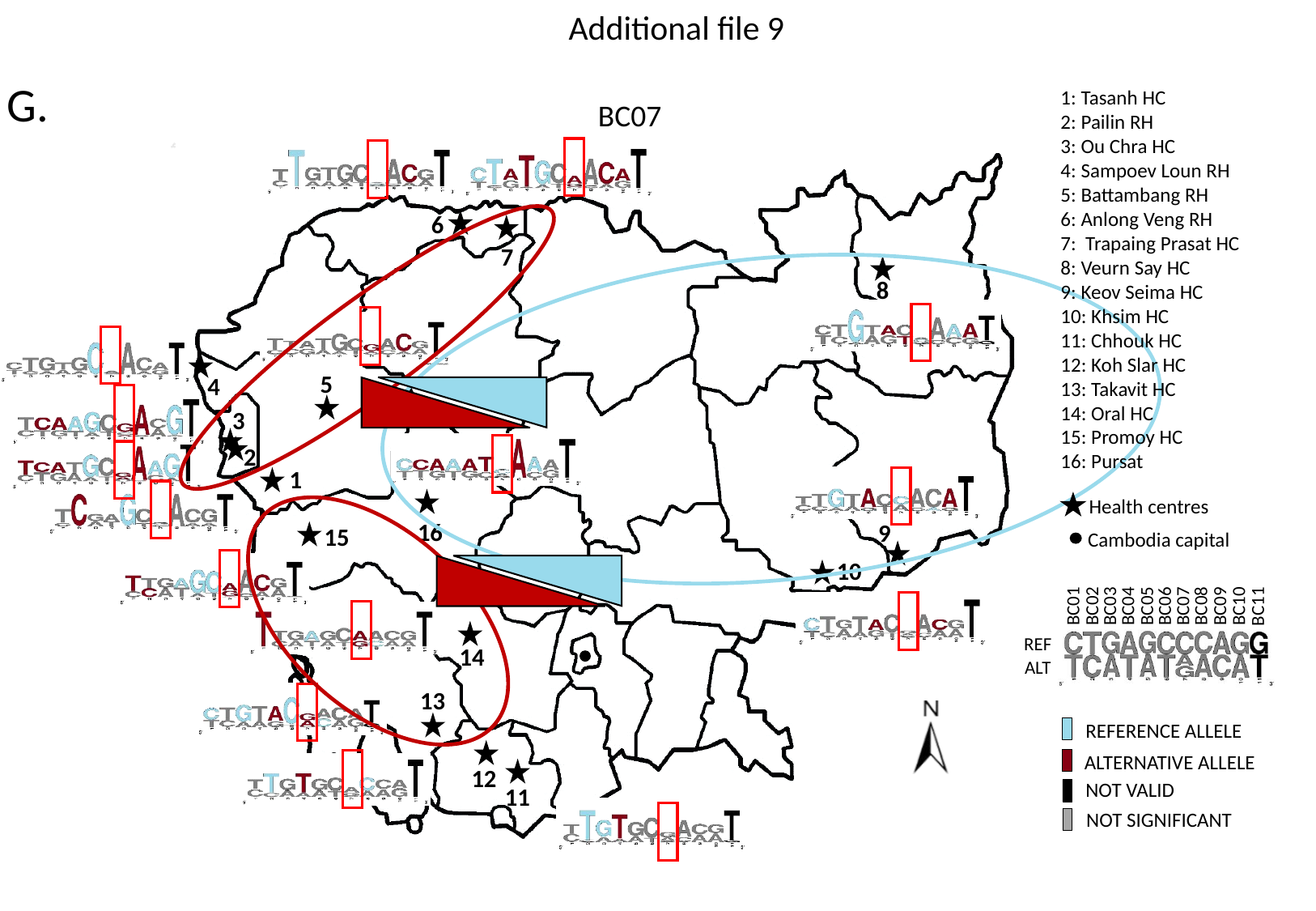

Additional file 9
G.
1: Tasanh HC
2: Pailin RH
3: Ou Chra HC
4: Sampoev Loun RH
5: Battambang RH
6: Anlong Veng RH
7: Trapaing Prasat HC
8: Veurn Say HC
9: Keov Seima HC
10: Khsim HC
11: Chhouk HC
12: Koh Slar HC
13: Takavit HC
14: Oral HC
15: Promoy HC
16: Pursat
BC07
6
7
8
5
4
3
2
1
16
9
15
10
14
13
12
11
Health centres
Cambodia capital
BC01
BC02
BC03
BC04
BC05
BC06
BC07
BC08
BC09
BC10
BC11
REF
ALT
REFERENCE ALLELE
ALTERNATIVE ALLELE
NOT VALID
NOT SIGNIFICANT

## Slide 8
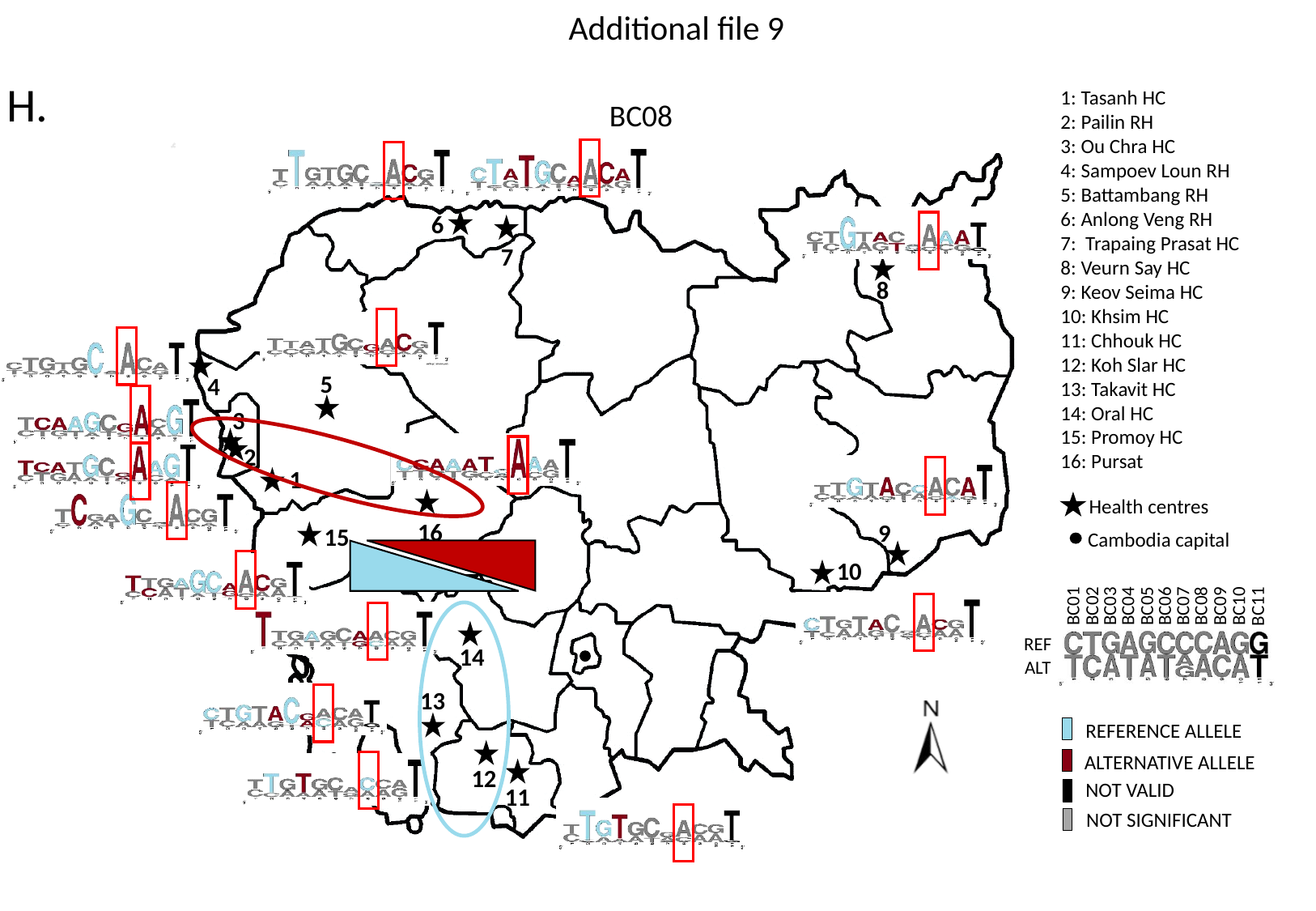

Additional file 9
H.
1: Tasanh HC
2: Pailin RH
3: Ou Chra HC
4: Sampoev Loun RH
5: Battambang RH
6: Anlong Veng RH
7: Trapaing Prasat HC
8: Veurn Say HC
9: Keov Seima HC
10: Khsim HC
11: Chhouk HC
12: Koh Slar HC
13: Takavit HC
14: Oral HC
15: Promoy HC
16: Pursat
BC08
6
7
8
5
4
3
2
1
16
9
15
10
14
13
12
11
Health centres
Cambodia capital
BC01
BC02
BC03
BC04
BC05
BC06
BC07
BC08
BC09
BC10
BC11
REF
ALT
REFERENCE ALLELE
ALTERNATIVE ALLELE
NOT VALID
NOT SIGNIFICANT

## Slide 9
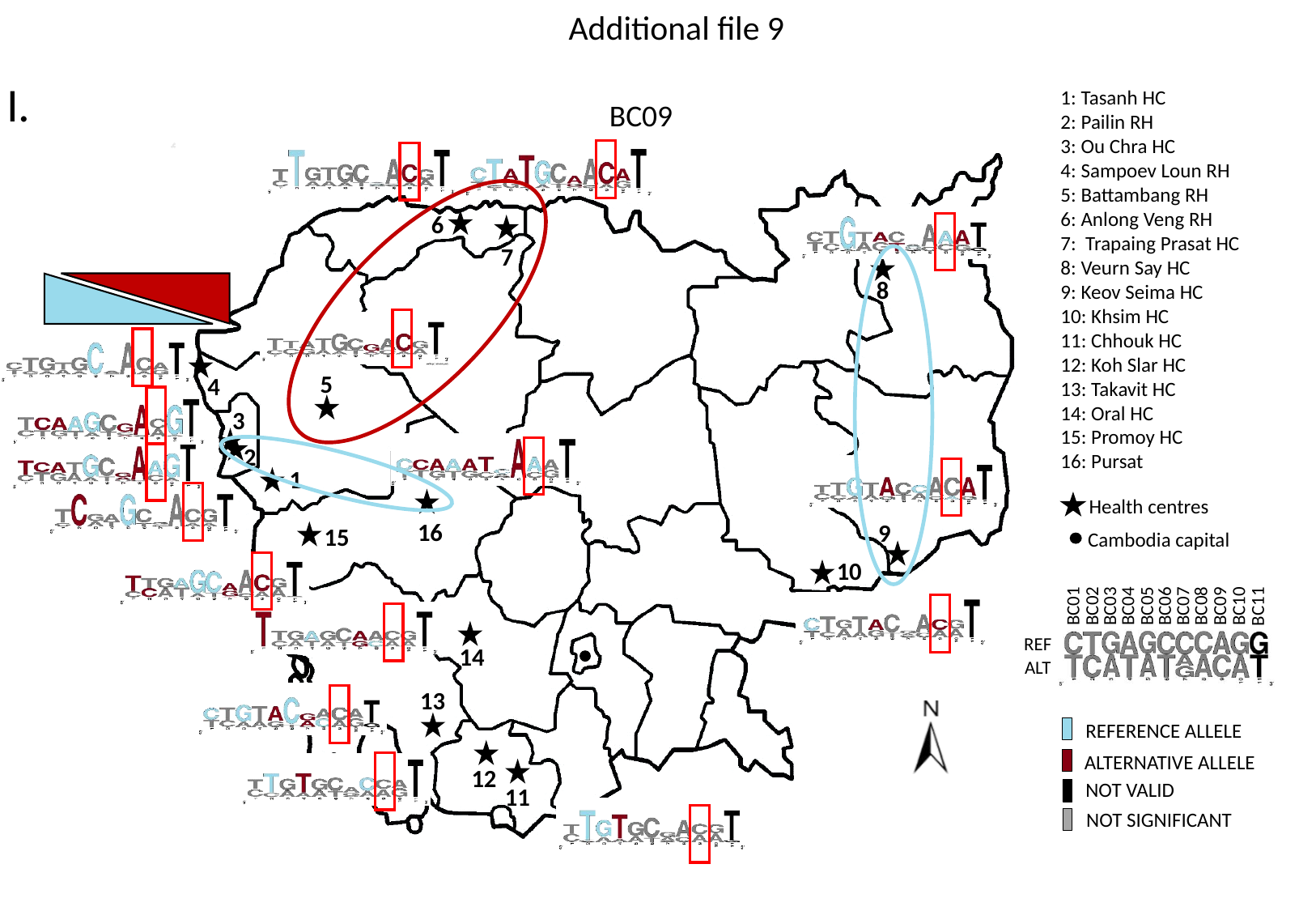

Additional file 9
I.
1: Tasanh HC
2: Pailin RH
3: Ou Chra HC
4: Sampoev Loun RH
5: Battambang RH
6: Anlong Veng RH
7: Trapaing Prasat HC
8: Veurn Say HC
9: Keov Seima HC
10: Khsim HC
11: Chhouk HC
12: Koh Slar HC
13: Takavit HC
14: Oral HC
15: Promoy HC
16: Pursat
BC09
6
7
8
5
4
3
2
1
16
9
15
10
14
13
12
11
Health centres
Cambodia capital
BC01
BC02
BC03
BC04
BC05
BC06
BC07
BC08
BC09
BC10
BC11
REF
ALT
REFERENCE ALLELE
ALTERNATIVE ALLELE
NOT VALID
NOT SIGNIFICANT

## Slide 10
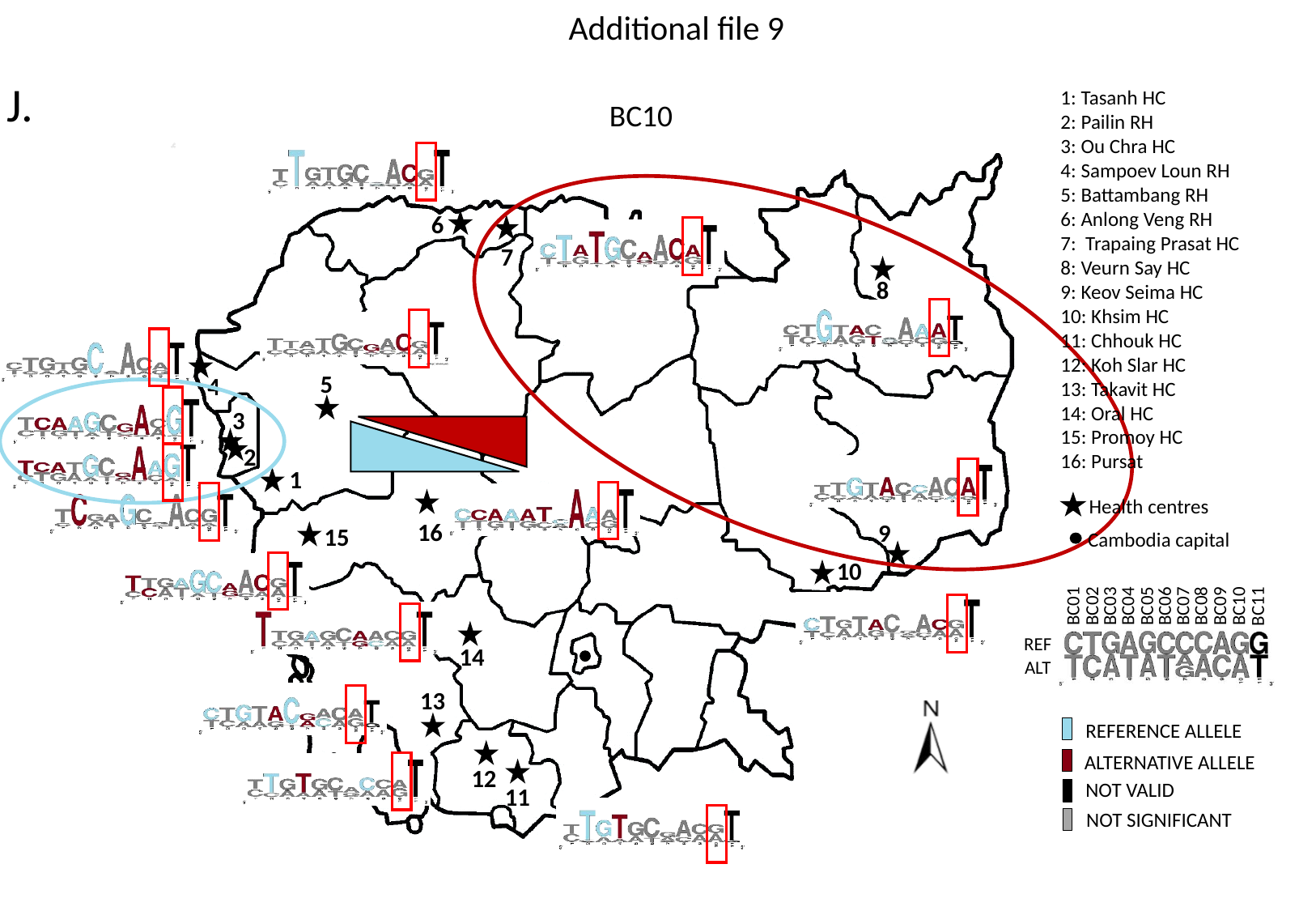

Additional file 9
J.
1: Tasanh HC
2: Pailin RH
3: Ou Chra HC
4: Sampoev Loun RH
5: Battambang RH
6: Anlong Veng RH
7: Trapaing Prasat HC
8: Veurn Say HC
9: Keov Seima HC
10: Khsim HC
11: Chhouk HC
12: Koh Slar HC
13: Takavit HC
14: Oral HC
15: Promoy HC
16: Pursat
BC10
6
7
8
5
4
3
2
1
16
9
15
10
14
13
12
11
Health centres
Cambodia capital
BC01
BC02
BC03
BC04
BC05
BC06
BC07
BC08
BC09
BC10
BC11
REF
ALT
REFERENCE ALLELE
ALTERNATIVE ALLELE
NOT VALID
NOT SIGNIFICANT
