# Supplementary material for: Plasmodium falciparum parasite population structure and gene flow associated to anti-malarial drugs resistance in Cambodia
Source: Malar J. 2016 Jun 14;15:319. doi: 10.1186/s12936-016-1370-y (PMC4908689; doi:10.1186/s12936-016-1370-y)
Supplement: Supplementary file 11 — 10.1186/s12936-016-1370-y Comparison of allele frequencies in health centre and in conserved clustering groups. A comparative analysis was illustrated using Weblogos sorted for health centre and clustering groups. [file 12936_2016_1370_MOESM11_ESM.pptx]

## Slide 1
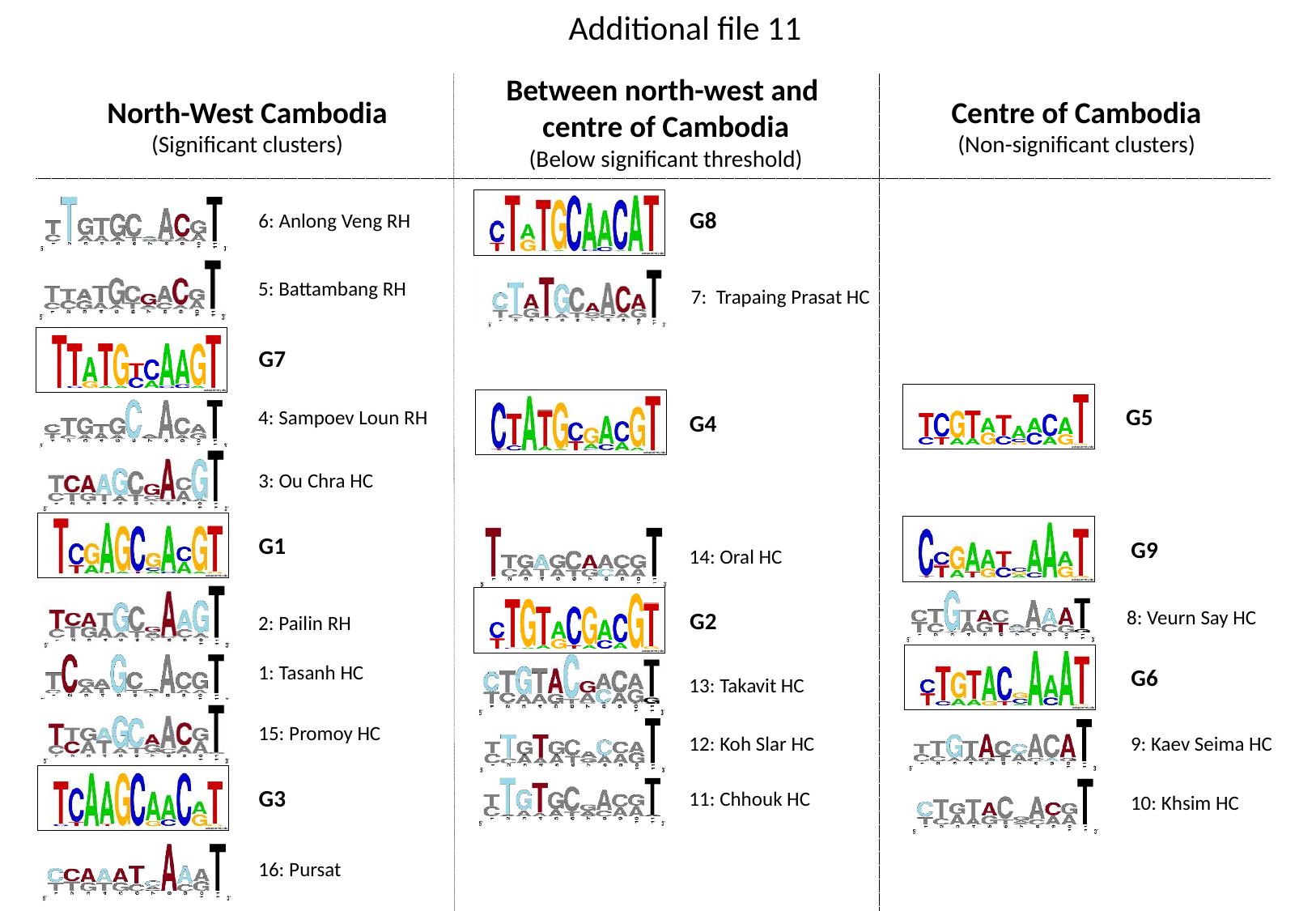

Additional file 11
Between north-west and
centre of Cambodia
(Below significant threshold)
Centre of Cambodia
(Non-significant clusters)
North-West Cambodia
(Significant clusters)
6: Anlong Veng RH
5: Battambang RH
G7
4: Sampoev Loun RH
3: Ou Chra HC
G1
2: Pailin RH
1: Tasanh HC
15: Promoy HC
G3
16: Pursat
G8
7: Trapaing Prasat HC
G4
14: Oral HC
G2
13: Takavit HC
12: Koh Slar HC
11: Chhouk HC
G5
G9
8: Veurn Say HC
G6
9: Kaev Seima HC
10: Khsim HC
