# Supplementary material for: Plasmodium falciparum parasite population structure and gene flow associated to anti-malarial drugs resistance in Cambodia
Source: Malar J. 2016 Jun 14;15:319. doi: 10.1186/s12936-016-1370-y (PMC4908689; doi:10.1186/s12936-016-1370-y)
Supplement: Supplementary file 12 — 10.1186/s12936-016-1370-y Distribution of IC50 value of P. falciparum isolates per major geographic areas. Box Plot analysis is presenting median and quartiles. Dashed line figure out the threshold where parasite could be resistant for the drug (30 nM). Parasites were originating from regions distributed at the four compass points in Cambodia. ANOVA test was significant for chloroquine and mefloquine (p value = 5.62e−5 and p value = 0.0408, respectively). [file 12936_2016_1370_MOESM12_ESM.pptx]

## Slide 1
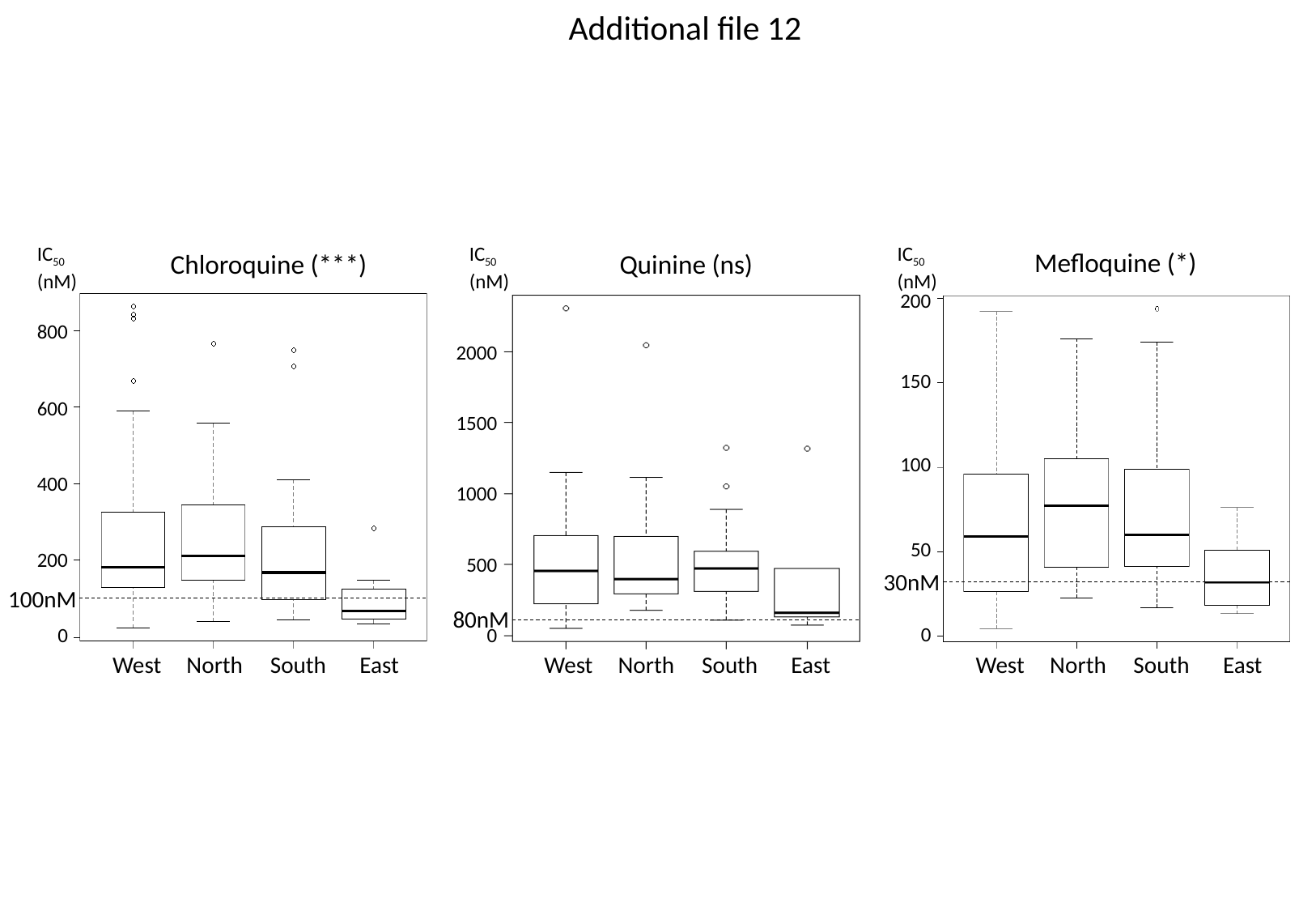

Additional file 12
IC50
(nM)
IC50
(nM)
IC50
(nM)
Mefloquine (*)
Quinine (ns)
Chloroquine (***)
200
150
100
50
0
800
600
400
200
0
2000
1500
1000
500
0
30nM
100nM
80nM
West
North
South
East
West
North
South
East
West
North
South
East
