# Supplementary material for: Plasmodium falciparum parasite population structure and gene flow associated to anti-malarial drugs resistance in Cambodia
Source: Malar J. 2016 Jun 14;15:319. doi: 10.1186/s12936-016-1370-y (PMC4908689; doi:10.1186/s12936-016-1370-y)
Supplement: Supplementary file 13 — 10.1186/s12936-016-1370-y Distribution of mefloquine IC50 value of isolates associated to conserved clusters G1 to G9. Box Plot analysis is presenting median and quartiles. Mefloquine resistant parasites have IC50 over the dashed line (30 nM). [file 12936_2016_1370_MOESM13_ESM.pptx]

## Slide 1
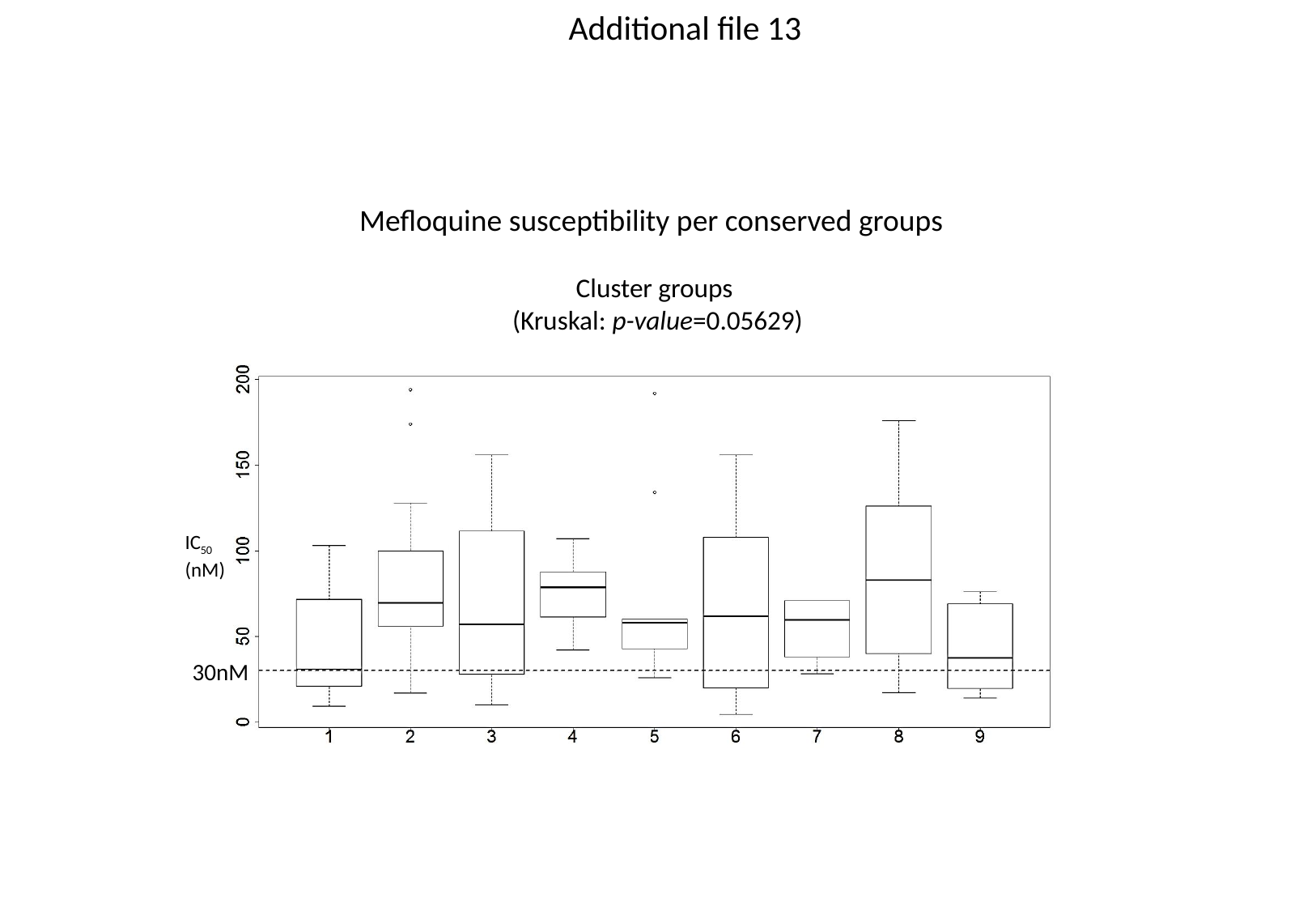

Additional file 13
Mefloquine susceptibility per conserved groups
Cluster groups
(Kruskal: p-value=0.05629)
IC50
(nM)
30nM
